# Gel‐Free Capture of Microvolt‐Level Neural Signatures Via a 3D Monolithic Carbon Fiber Dry Electrode

**DOI:** 10.1002/advs.78531

**Published:** 2026-10-29

**Authors:** Haoxuan Sun, Nikolay Novitskiy, Dongwook Jang, Yixiang Liu, Shaoqi Pan, Ting Huang, Yitong Li, Huisi Yang, Leonardo Nicholas Adi Wijaya, Md Masruck Alam, Patrick CM Wong, Zhaoli Gao

**Affiliations:** ^1^ Department of Biomedical Engineering The Chinese University of Hong Kong Hong Kong SAR China; ^2^ Brain and Mind Institute The Chinese University of Hong Kong Hong Kong SAR China; ^3^ Department of Linguistics and Modern Languages The Chinese University of Hong Kong Hong Kong SAR China; ^4^ Department of Psychology The Chinese University of Hong Kong Hong Kong SAR China

**Keywords:** biomedical engineering, electrical impedance, electrode, electrophysiology, neurophysiology

## Abstract

Gel‐based wet electrodes remain the gold standard for electrophysiology, but gel dehydration limits recordings. Existing dry electrodes partially address this limitation but suffer from high interfacial impedance. Here, a carbon fiber‐based porous electrode (CFPE) is reported, comprising a three‐dimensional conductive network coated with poly(3,4‐ethylenedioxythiophene):poly(styrene sulfonate) (PEDOT:PSS). The CFPE establishes contact at hairy‐scalp sites with an impedance of approximately 10 kΩ·cm^2^. On the forearm, its impedance at 10 Hz without skin preparation is 11.09 ± 1.03 kΩ·cm^2^, compared with 13.57 ± 2.07 kΩ·cm^2^ for wet electrodes. After 6 h of wear, CFPE impedance reaches 1.3 times its initial value without skin preparation or gel, whereas wet‐electrode impedance reaches 56 times its initial value when applied with skin preparation and conductive gel. High‐fidelity electrocardiography, electromyography, and electroencephalography signals are captured with correlation coefficients exceeding 90% and signal‐to‐noise ratios comparable to wet electrodes. The CFPE further resolves microvolt‐level speech‐evoked frequency‐following responses (FFR) and long‐latency responses (LLR), with fidelity comparable to wet electrodes and significantly higher LLR SNR. These results demonstrate gel‐free acquisition of weak neural biomarkers, opening a pathway toward prolonged, preparation‐free neurophysiology.

## Graphical abstract

A compressible carbon fiber/PEDOT:PSS electrode enables gel‐free contact at hairy scalp sites. Its conductive porous framework supports low interfacial impedance and retains contact performance after repeated mechanical and sweat‐related challenges. Beyond routine electrophysiological recording, the electrode captures microvolt‐level speech‐evoked frequency‐following and long‐latency responses, highlighting its potential for gel‐free recording of weak neural signals.

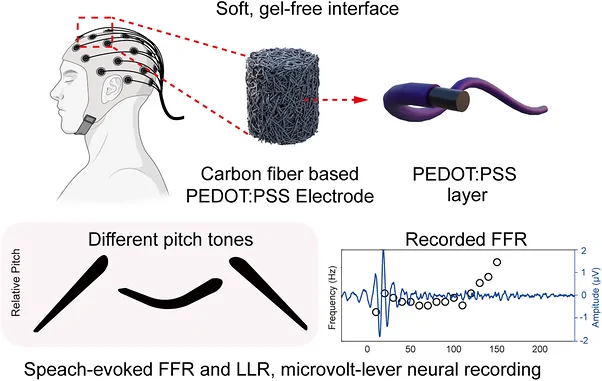

## 1 Introduction

Electrophysiological signals [1, 2, 3], ranging from cardiac rhythms (ECG) [4] and muscle activity (EMG) [5] to neural oscillations (EEG) [6], are indispensable for clinical diagnostics, rehabilitation, and brain‐computer interfaces. The fidelity of these recordings is governed by the electrode‐skin interface [7]. Wet Ag/AgCl electrodes remain the gold standard [8]; however, their reliance on conductive gels introduces a fundamental limitation: gel dehydration over hours leads to progressive impedance escalation and signal degradation [9], while the requirements for skin abrasion and post‐recording cleanup restrict applicability in continuous and home‐based monitoring [10, 11]. The inability of wet electrodes to maintain stable impedance over extended periods represents a critical barrier to prolonged electrophysiology. Significant progress has been made in gel‐free dry electrodes [12, 13], including sponge [14, 15], textile‐ [16, 17], elastomer‐ [18, 19], and microneedle‐ [20, 21] based designs. However, most flexible dry electrodes rely on non‐conductive substrates [14, 22] with thin conductive surface coatings [14], resulting in high interfacial impedance on irregular or hairy skin. As a result, existing dry electrodes remain largely confined to routine biopotential monitoring and, to our knowledge, have not demonstrated the capacity to resolve microvolt‐level neural signatures such as speech‐evoked frequency‐following responses (FFR), which require exceptionally low and stable electrode‐skin impedance over thousands of stimulus presentations.

Here, we report a carbon fiber‐based porous electrode (CFPE) comprising a three‐dimensional monolithic conductive network of carbon fibers coated with poly(3,4‐ethylenedioxythiophene):poly(styrene sulfonate) (PEDOT:PSS). Unlike electrodes that rely on a conductive coating over an insulating scaffold, the framework provides conductive pathways throughout the electrode body, so that similar contact impedance was observed across the tested electrode thicknesses and is retained despite localized coating cracks. The resulting 10 mm‐thick, compressible electrode establishes gel‐free contact at hairy‐scalp sites under a standard EEG cap and enables the acquisition of speech‐evoked FFR, which, to our knowledge, has not been achieved with a dry electrode before. The compressible fibrous architecture establishes direct, low‐impedance contact with the skin (11.09 ± 1.03 kΩ·cm^2^ at 10 Hz) without any skin preparation and maintains stable impedance over 6 h of continuous wear while wet electrodes, applied with standard skin preparation, degrade by 56‐fold. We demonstrate high‐fidelity signal acquisition across ECG, EMG, and EEG (correlation coefficient >90%), with the electrode exhibiting exceptional durability over 10 000 compression cycles and 8 weeks of storage. To assess the ability of this platform, microvolt‐level FFR and LLR signals were captured from adult subjects, achieving signal fidelity comparable to wet electrodes.

## 2 Results and Discussion

We developed carbon fiber/poly(3,4‐ethylenedioxythiophene):poly(styrene sulfonate) electrodes (CFPEs), comprising a three‐dimensional monolithic conductive network of carbon fibers coated with a PEDOT:PSS transduction layer. Figure 1a illustrates the fabrication process. Polyacrylonitrile (PAN) fiber cubes were first carbonized to form the conductive scaffold; however, the resulting high carbon content (>90%) leads to poor wettability, which restricts effective infiltration of the PEDOT:PSS solution [23]. To address this, the carbon fibers were modified with oxygen‐containing functional groups via electrochemical oxidation to construct an active interface [24], thereby enhancing compatibility with the PEDOT:PSS matrix.

**FIGURE 1:**
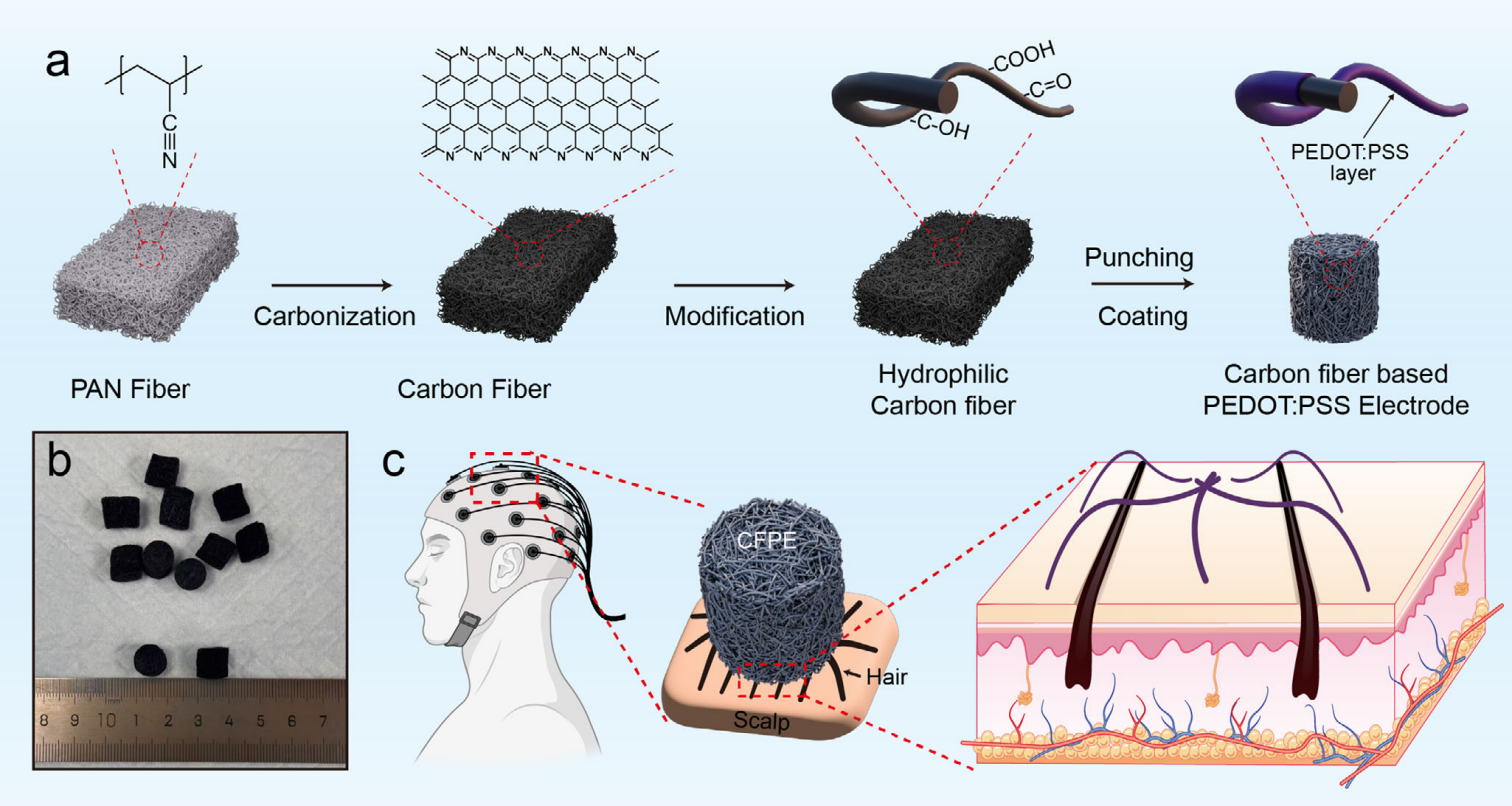
(a) Schematic illustrations of the CFPEs fabrication process, which includes carbonization, modification, and punching. (b) Photograph of CFPEs. (c) A schematic illustration of the carbon fiber‐based electrodes integrated on a commercial EEG hat to achieve conformal contact with the hairy scalp.

The modified cubes were then punched to ensure dimensional uniformity, dip‐coated in PEDOT:PSS, and annealed to form stable composite electrodes. The resulting CFPEs (10 mm diameter and height, Figure 1b) feature a customizable geometry that facilitates direct integration with commercial EEG systems. As shown in Figure 1c, the fibrous architecture conforms to the multiscale topography of the hairy scalp, effectively minimizing the air gap at the electrode‐scalp interface.

To verify the effectiveness of key fabrication steps (i.e., PAN fiber carbonization and carbon fiber cube (CFC) modification), comprehensive characterization was conducted. Figure 2a presents the Raman spectra of PAN fibers before and after carbonization. The pristine PAN fiber (black curve) exhibits no distinct Raman peaks, which is typical of its amorphous polymeric nature. In contrast, the carbonized PAN fiber (red curve) displays the disorder‐induced D band at 1352 cm^−^^1^ and the graphitic G band at 1575 cm^−^^1^, both characteristic of carbon materials [25], confirming the successful conversion of PAN fibers to carbon fibers.

**FIGURE 2:**
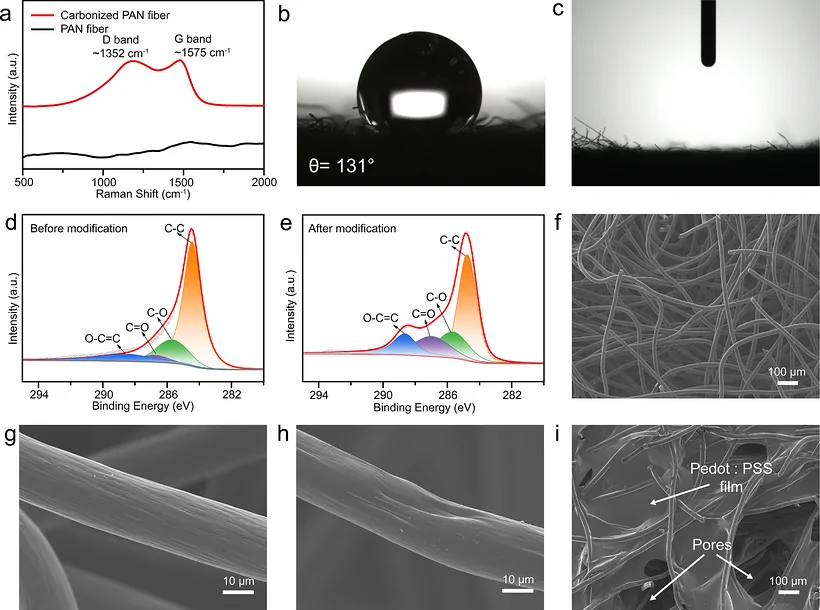
(a) The Raman spectra of PAN fibers, before and after carbonization. Static contact angle measurement of CFC (b) before and (c) after modification. C1s XPS spectra of carbon fiber cube (d) before and (e) after modification. (f) SEM images of pristine CFC. Enlarged SEM images (g) before and (h) after modification. (i) SEM images of CFC after PEDOT:PSS coating.

The carbonization process endows the CFC with both excellent conductivity and strong hydrophobic behavior, evidenced by a water contact angle of 131° (Figure 2b), which is attributed to the reduced number of surface polar functional groups and increased carbon content. Electrochemical oxidation in sulfuric acid was employed to modify the CFC surface, generating oxygen‐containing functional groups (e.g., ‐OH, C = O, ‐COOH) on the carbon fiber surface, which promotes both wettability and interfacial adhesion with the PEDOT:PSS layer. The modified CFC completely absorbs a 10 µL water droplet within 1 second (Figure 2c). Consistent with this improved wettability, unmodified CFCs floated on deionized water, whereas modified CFCs sank after immersion (Figure S1). This enhanced hydrophilicity allows the rapid and complete immersion of the treated CFC into the PEDOT:PSS solution, while also promoting stronger interfacial bonding between the carbon fiber skeleton and the PEDOT:PSS layer.

X‐ray photoelectron spectroscopy (XPS) was utilized to investigate the changes in surface states on carbon fibers before and after oxidation treatment (Figure 2d, e). The CFC surface contained four carbon species: C‐C, C‐O, C = O, and ‐COOH, with binding energy positions consistent with reported values at 284.6 eV, 285.7 eV, 287.0 eV, and 288.8 eV, respectively [26, 27]. After modification, the peak areas of carbonyl (C = O) and carboxyl (‐COOH) groups increased significantly, and the oxygen content on the CFC surface increased from 7.65% to 27.63% (Table 1), corresponding to a substantially higher density of active bonding sites for PEDOT:PSS adhesion. Scanning electron microscopy (SEM) images further reveal the structural changes during fabrication. The CFC possessed an entangled fiber network (Figure 2f) with relatively smooth individual carbon fiber surfaces (Figure 2g). After modification, obvious microscale grooves appeared on the fibers (Figure 2h **),** formed by the electrochemical etching effect during oxidation. This structural change increases the specific surface area of the carbon fibers, providing more attachment sites for PEDOT:PSS coating and enhancing mechanical interlocking between the two materials. After PEDOT:PSS coating, the fiber surfaces become uniformly coated, and a PEDOT:PSS interconnection structure was formed between fibers (Figure 2i).

**TABLE 1:** Summary of element composition on carbon fiber surface.

Sample	Relative contents of chemical element (%)		Atomic ratio (O/C)
	C	O	
Before modification	92.35	7.65	0.083
After modification	72.37	27.63	0.382

Energy dispersive X‐ray spectroscopy (EDX) results (Figure S2) confirm that carbon, oxygen, and sulfur elements are evenly distributed across the fiber surface, verifying the formation of a three‐dimensional integrated PEDOT:PSS‐carbon fiber conductive structure. In CFPE, the carbonized‐fiber framework is itself the conductor and the compressible support, and PEDOT:PSS coats and bridges the surface‐functionalized fibers to modify the electrical interface. Unlike electrodes that rely on a conductive coating over an insulating scaffold, such as carbon‐fiber‐filled elastomer sponges and PEDOT:PSS‐coated polymer sponges, the framework provides conductive pathways throughout the electrode body, so that contact performance is retained despite localized coating cracks after cyclic compression (Figure S7). The pressure‐dependent impedance measurements are consistent with improved electrical coupling upon compression (Figure S5), while the contact‐impedance measurements after cyclic compression and repeated application/removal support retention of contact performance after repeated mechanical loading (Figure S8 and Table S3).

For practical wearable application, dry electrodes must withstand repeated compression, resist NaCl corrosion, and maintain performance over weeks of storage. The porous architecture and fabrication process impart significant compressibility to the CFPEs, and the mechanical properties under 70% compressive strain with different coating (immersion) cycles were investigated. As shown in Figure 3a, under low strain conditions, the stress of CFPEs changes slightly; however, as the strain increases, the stress rises rapidly, which is attributed to the gradual compression of internal pore spaces. CFPEs under low coating cycles (0‐2 cycles) exhibit similar stress‐strain curves with compressive stress of approximately 1.96, 3.46 and 9.42 kPa at 10% compression, respectively (Figure S3). In contrast, after 4 coating cycles, CFPEs generate significant stress even under small strain, which is attributed to the accumulation of PEDOT:PSS that fills most of the inter‐fiber spaces. The electrical properties can also be regulated by the number of coating cycles. The resistance of the uncoated carbon fiber cubes (CFCs; 0 coating cycles) was 14.37 ± 0.95 Ω, measured between two probe contacts separated by the sample thickness of 10 mm. After one coating cycle, the resistance decreased to 1.61 ± 0.09 Ω (Figure 3b), with little further reduction upon additional coating cycles, which is attributed to the uniform formation of continuous conductive pathways by PEDOT:PSS on the carbon fiber surfaces.

**FIGURE 3:**
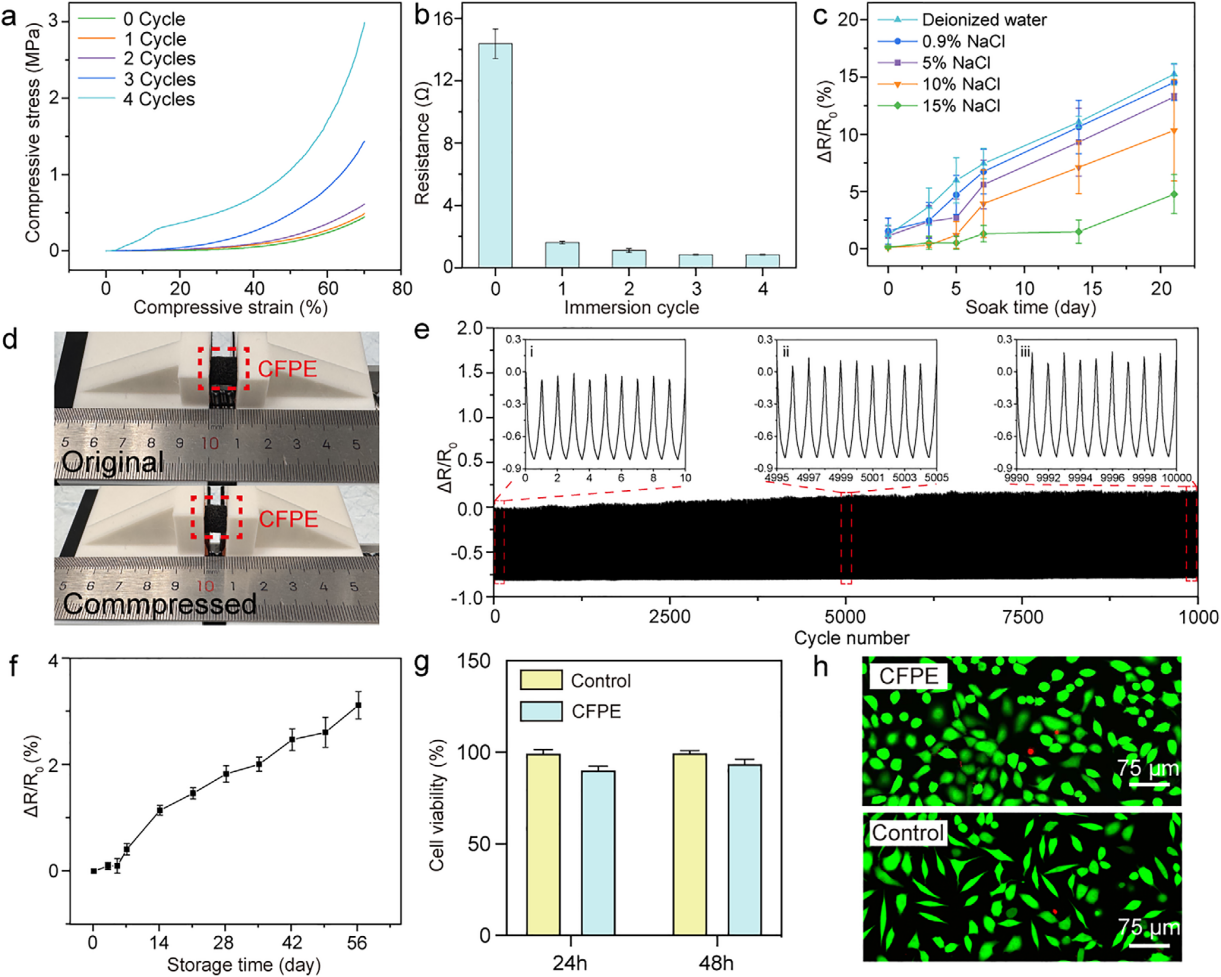
(a) Stress‐strain curve of CFPEs with different immersion cycles recorded under 70% maximum compressive strains. (b) Resistance of CFPEs fabricated with different immersion cycle numbers. (c) The resistance changes over 21 days in different concentrations of NaCl solutions. (d) Images of the mechanical stability test setup for the CFPEs in the initial state and at 15% compressive strain. (e) The real‐time resistance‐change recording for the 15% compression ratio experiments for 10 000 cycles and (i) the first ten cycles, (ii) the middle ten cycles, and (iii) the last ten cycles. (f) The resistance changes over 8 weeks in a sealed bag. (g) L929 cell viability after exposure to CFPE extracts for 24 and 48 h, assessed using the CCK‐8 assay. Data are presented as mean ± SD from six replicate wells per condition at each time point within a single experiment (n = 6 wells). (h) Representative fluorescence images of L929 cells after 48 h of exposure to CFPE extracts (top) and control medium (bottom). Green fluorescence represents live cells, whereas red indicates dead cells. Data in panels b, c and f are presented as mean ± SD (n = 6 electrodes per condition).

To evaluate the conductive stability of CFPEs under daily use environments, the resistance changes in NaCl solutions of different concentrations (0%, 0.9%, 5%, 10%, and 15%) were measured (Figure 3c). When immersed in 0.9% NaCl solution for 21 days, the resistance only increased by approximately 14%, confirming long‐term stability against NaCl corrosion. The cyclic mechanical stability was tested through repeated compression experiments (Figure 3d). After 10,000 cycles of compression with a 15% compression ratio, the compressed‐state resistance remained stable, while the uncompressed resistance increased by approximately 15% (Figure 3e), which is attributed to irreversible microstructural deformation that does not affect practical use under applied pressure. The long‐term storage stability was also validated. After 8 weeks in sealed bags, the resistance increased by only approximately 3% (Figure 3f), which is attributed to the long‐term structural stability of the carbon fiber skeleton and the stable interface formed between the annealed PEDOT:PSS film and the carbon fibers. To verify the biocompatibility of CFPEs, their potential cytotoxicity was evaluated using L929 cells based on the ISO 10993 standards. The viability of L929 cells cultured in medium containing CFPEs extracts exceeded 90% at both 24 h and 48 h post‐incubation, indicating no substantial reduction in cell viability under the tested extraction and exposure conditions (Figure 3g). Fluorescence microscopy images further confirmed that the morphology and density of viable cells in the CFPEs group were comparable to those in the blank control group (Figure 3h). Beyond the in vitro cytotoxicity assessment, the biological response to extracts from the CFPE was independently evaluated using skin irritation and sensitization tests. Polar and non‐polar CFPE extracts were prepared using 0.9% sodium chloride injection solution and sesame oil, respectively. In the multiple‐contact rabbit skin irritation test, neither extract caused detectable erythema or edema, with a cumulative irritation index of 0. Skin sensitization was further assessed using the Guinea Pig Maximization Test (GPMT). Both polar and non‐polar CFPE extracts showed a sensitization incidence of 0%, while the positive control showed a 100% incidence. These findings provide evidence supporting the biocompatibility of the CFPE. Detailed test conditions and results are provided in Table S1.

Equivalent‐circuit fitting was used to quantitatively characterize the electrode‐skin impedance of the wet electrode and CFPE (Figure 4a and Figure S4). The wet electrode was fitted using R1 + (CPE || R2), whereas CFPE was fitted using R1 + [CPE || (R2 + W)]. R1 represents the lumped ohmic resistance, R2 describes an effective interfacial resistive contribution, and the constant phase element (CPE) accounts for non‐ideal capacitive behavior. The additional Warburg element (W) in the CFPE model represents a diffusion‐related impedance contribution. The fitted R1 values were 48.9 and 316.6 Ω·cm^2^ for CFPE and the wet electrode, respectively, indicating a lower effective ohmic contribution for CFPE. The corresponding model‐specific R2 values were 1,337 and 17,435 Ω·cm^2^, with the complete fitting parameters summarized in Table S2.

**FIGURE 4:**
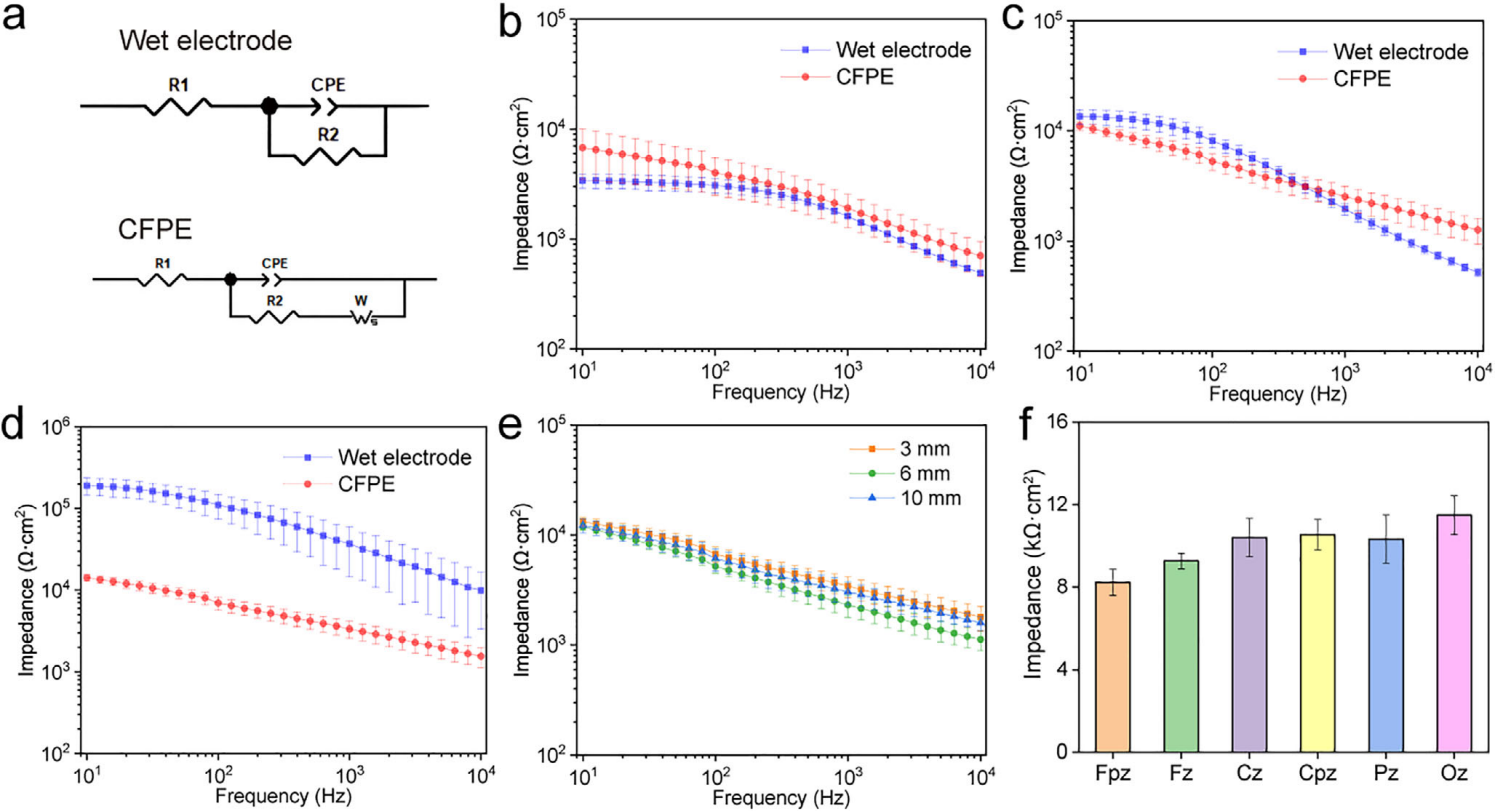
(a) Equivalent‐circuit models used to fit the EIS spectra of the wet‐electrode‐skin and CFPE‐skin interfaces. (b) Impedance spectrum of wet electrodes and CFPE measured with skin preparation. (c) Impedance spectrum of wet electrodes and CFPE measured without skin preparation. (d) Impedance spectra of the wet electrode and the CFPE after 6 h of continuous wear. The wet electrode was applied with skin preparation and fresh conductive gel; the CFPE was applied without skin preparation. (e) Impedance spectra acquired by CFPEs of different thickness. (f) The contact impedance of CFPEs on different scalp locations. Data in panels b‐f are presented as mean ± SD (n = 3 participants per condition).

To evaluate the impedance performance of the CFPE, its contact impedance was benchmarked against commercial Ag/AgCl wet electrodes. With skin preparation, the wet electrode exhibits lower impedance (3.40 ± 0.50 kΩ·cm^2^) due to the high ionic conductivity of the gel, while the CFPE achieves a comparable impedance profile (6.36 ± 3.20 kΩ·cm^2^, Figure 4b). To evaluate the stability of the electrode‐skin interface during wear, we recorded the impedance changes over 6 h of continuous wearing (Figure 4c,d). Each electrode was applied as it is intended to be used: the commercial wet electrode with skin preparation and freshly applied conductive gel, following standard practice for wet electrodes, and the CFPE without any skin preparation or gel. In the absence of skin pre‐treatment (Figure 4c), the CFPE maintains a lower impedance (11.09 ± 1.03 kΩ·cm^2^) than the wet electrode (13.57 ± 2.07 kΩ·cm^2^). This result supports effective electrode‐skin coupling without abrasive skin preparation under the tested conditions. The most critical limitation of conventional wet electrodes resides in their reliance on the aqueous gel, which gradually evaporates over time. As demonstrated in

Figure 4d, after 6 h of continuous wear, the impedance of the commercial wet electrode increased by approximately 56‐fold, from 3.40 ± 0.50 to 190.81 ± 44.68 kΩ·cm^2^, despite having been applied with skin preparation and fresh conductive gel. In contrast, the CFPE impedance increased by only 1.3‐fold, from 11.09 ± 1.03 to 14.20 ± 4.68 kΩ·cm^2^, demonstrating that the gel‐free architecture eliminates the primary failure mode of conventional electrodes.

Beyond the 6 h wear test, we evaluated CFPEs under use‐relevant mechanical and environmental challenges at the electrode‐skin interface. CFPEs were subjected to 8 000 cyclic compressions on a skin‐mimicking model (Figure S6), followed by contact‐impedance measurement and SEM characterization. The 10 Hz contact impedance changed from 11.69 ± 0.70 kΩ·cm^2^ for fresh CFPEs to 12.13 ± 1.20 and 12.77 ± 0.29 kΩ·cm^2^ after 4,000 and 8,000 cycles, respectively (Table S3), and SEM images after 8,000 cycles showed minor localized cracking of the PEDOT:PSS layer but no widespread delamination or disruption of the fibrous network (Figure S7). Because the CFPE is intended as a replaceable, cap‐ or strap‐assisted electrode module rather than a self‐adhesive patch, its repeated‐use contact stability was assessed by 100 application/removal cycles; the 10 Hz contact impedance was 12.11 ± 0.28 kΩ·cm^2^ after 50 cycles and 12.56 ± 0.22 kΩ·cm^2^ after 100 cycles (Table S3). The CFPEs were also exposed to 50 artificial‐sweat wet‐dry cycles at pH 4.7, 5.5 and 6.5, after which the 10 Hz contact impedance remained between 11.9 and 12.5 kΩ·cm^2^ (Table S3). At the final measurement point of each durability test, the mean 10 Hz contact impedance remained within 10% of the fresh‐electrode reference value. The retention of contact performance after cyclic compression, repeated application‐removal cycles, and artificial‐sweat exposure, together with the small impedance drift observed after 6 h of wear, indicates that CFPEs are potentially suitable for repeated use.

For practical wearable use, dimensional versatility and hair compatibility are crucial. Contact impedance tests were conducted on CFPEs with three different thicknesses (3, 6, and 10 mm) on the forearm under a pressure range from 3.01 to 7.25 kPa. As shown in Figure 4e, all three thicknesses exhibited low contact impedance with no correlation between electrode thickness and impedance, confirming that CFPEs can be fabricated into either planar or three‐dimensional geometries without sacrificing conductivity. When integrated into a commercial EEG cap (Greentek), the contact impedance at the hairless forehead position (Fpz) was 8.23 ± 0.65 kΩ·cm^2^, while at hairy positions, the impedance remained around 10 kΩ·cm^2^ (Figure 4f), confirming that the compressible fibrous architecture effectively bypasses the macroscopic dielectric barrier of hair and maintains effective scalp contact at the tested hairy‐scalp sites.

To validate the reliability of CFPEs in practical electrophysiological recording, their performance was compared with that of commercial wet electrodes across ECG, EMG, and EEG. These three physiological signals here were all acquired using an OpenBCI Cyton board. CFPEs with a diameter of 10 mm and thickness of 2 mm were used for ECG and EMG, while CFPEs with a thickness of 10 mm were employed for EEG recordings. For ECG measurements, CFPEs and commercial wet electrodes were placed in close proximity to each other on the left arm, sharing the same reference and ground electrodes (Figure S9), allowing for simultaneous data acquisition. Both electrodes captured ECG signals with distinct PQRST waves (Figure 5a), with a correlation coefficient of 95.32% (Figure S10), the detailed calculation procedures of the correlation coefficient are provided in the Materials and Methods section. The signal‐to‐noise ratio (SNR) was 23.25 ± 0.74 dB for wet electrodes and 21.23 ± 0.41 dB for CFPEs (Table S4). In EMG experiments, CFPEs and commercial wet electrodes were simultaneously attached to the flexor carpi muscle on the right forearm (Figure S11). EMG signals were recorded while the subject gripped a hand gripper at three force levels: 10 N, 30 N, and 50 N. As shown in Figure 5b, higher grip forces induced correspondingly larger signal amplitudes, consistent with basic EMG physiological principles, with a 96.50% correlation coefficient between the two electrode types (Figure S12). The SNR of CFPEs (34.76 ± 2.23 dB) exceeded that of wet electrodes (31.10 ± 2.38 dB, Table S4). The active grip protocol, during which the forearm musculature undergoes substantial contraction and displacement, represents a mechanically demanding scenario for electrode‐skin coupling. Higher EMG amplitude was observed with CFPEs across the tested grip‐force levels (Figure S13).

**FIGURE 5:**
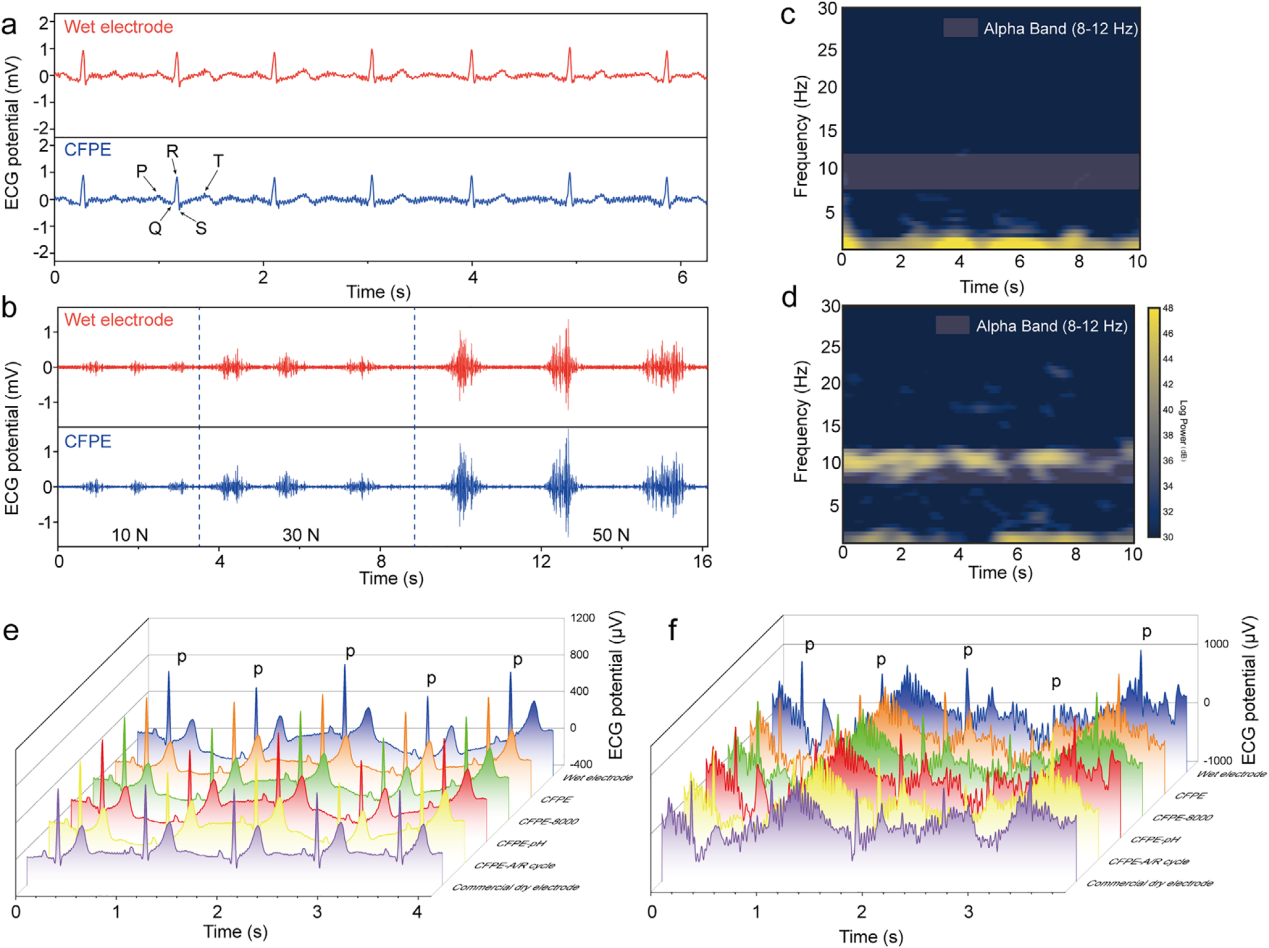
(a) Comparison of ECG signals using CFPEs and commercial wet electrode. (b) Comparison of EMG signals while gripping with different force levels. The time‐frequency spectrum (0‐30 Hz) of (c) eyes‐open and (d) eyes‐close periods. Representative ECG recordings obtained with different electrode conditions during (e) seated rest and (f) slow walking (3.6 km h^−^^1^).

EEG signal acquisition was performed using the 10 mm thick CFPEs, which conform to the multiscale topography of the hairy scalp to establish direct scalp contact under the pressure of the EEG cap. CFPEs and commercial wet electrodes were placed at positions O1 and O2, respectively, following the international 10–20 system (Figure S14). The EEG waveforms captured by CFPEs were highly consistent with those from wet electrodes, with a correlation coefficient of 91.7% (Figure S15). The SNR was 13.07 ± 1.39 dB for wet electrodes and 11.58 ± 2.19 dB for CFPEs (Table S4); a modest SNR difference of approximately 1.5 dB was observed under this recording configuration. As shown in Figure 5c, d, CFPEs captured the expected enhancement of alpha‐band (8‐12 Hz) activity during eyes‐closed compared with eyes‐open periods; the corresponding PSD comparison is provided in Figure S16, supporting the reliable recording of physiologically meaningful EEG activity.

To assess whether the durability tests affected the electrodes’ signal‐recording capability and to evaluate their dynamic ECG recording performance during motion, ECG was recorded at seated rest and during slow walking (3.6 km h^−^^1^) using fresh electrodes and CFPEs after the durability tests. Representative ECG waveforms are shown in Figure 5e,,f, with R peaks indicated. Although motion‐related waveform fluctuations were observed for all electrode types during walking, discernible R peaks were retained in recordings obtained with both fresh and durability‐challenged CFPEs. For descriptive quantification, the 0.05–0.5 Hz component of the ECG recordings was extracted, and RMS values are summarized in Table S5. These descriptive observations suggest similar ECG recording capability between CFPEs and wet electrodes under the tested slow‐walking condition, with identifiable R peaks retained after the durability challenges. These observations are promising for future application in ambulatory monitoring, where more demanding conditions such as head motion and running will further test artifact suppression.

Beyond routine biopotential monitoring, we evaluated the ability of CFPEs to capture ultra‐weak, high‐precision neurophysiological signatures. We specifically target the Frequency‐Following Response (FFR), a scalp‐recorded neurophonic potential in response to periodic auditory stimuli with the brainstem being its predominant source [28]. In the same auditory‐evoked paradigm, the cortical long‐latency response (LLR, also known as auditory evoked potential, AEP) can also be elicited. The FFR has been successfully used in the prediction of language development in infants even before they produce their first words [29, 30]. Since FFR and LLR signals are typically in the microvolt range, their acquisition traditionally demands wet electrodes and stringent skin preparation to ensure high signal‐to‐noise ratios (SNR).

We recorded EEG from 9 adults in the age range of 20–49 years with a Grael amplifier and Curry 9 software (Compumedics, Australia) using a sampling frequency of 4096 Hz. The subjects were seated in a comfortable chair while a sequence of 1998 Chinese syllables was delivered twice to their ears via insert headphones (Etymotic, USA). The syllables ’ga’ embedded one of three Chinese lexical tones, equiprobably: Cantonese Tone 2 (rising), Cantonese Tone 4 (low falling), or Mandarin Tone 3 (dipping). The electrode montage consisted of 3 working electrodes (Cz, left mastoid LM, and right mastoid RM), reference (CPz), and ground (Fpz) (Figure 6a).

**FIGURE 6:**
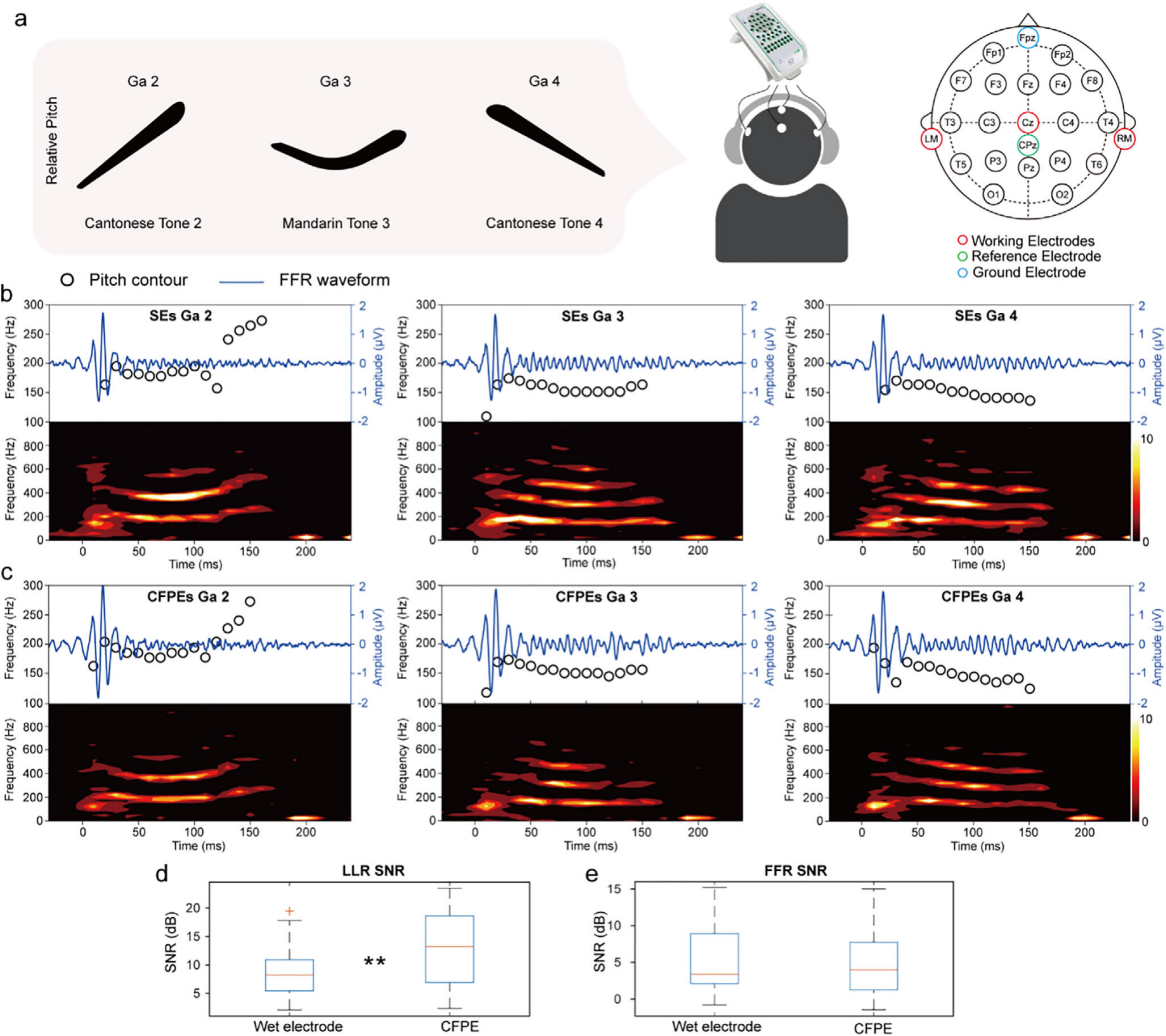
(a) Illustration of the three lexical tones and the electrode configuration used for FFR recordings. (b) standard Ag/AgCl electrodes (SEs) and (c) CFPEs as illustrated by their averaged waveform, median pitch contour (black circles) and median ITPC TFR (hot color). Pitch contours were extracted as the frequency of a maximum autocorrelation in every 40‐ms window of the time‐frequency representation of autocorrelation. ITPC is the vector average of the magnitude‐normalized complex FFT values that captures the degree of phase locking between the individual responses to the same sound. ITPC time‐frequency representation (TR) is produced by calculating FFT in 40‐ms overlapping time windows. The resulting TR was squared and multiplied by the number of individual trials. (d) LLR SNR and (e) FFR SNR recorded using wet electrodes and CFPEs. Statistical comparisons were performed using two‐tailed paired t‐tests (df = 47), with Benjamini‐Hochberg FDR correction across all 18 FFR/LLR features. BH‐adjusted P = 0.0044 for LLR SNR and 0.9620 for FFR SNR. **BH‐adjusted P < 0.01; ns, not significant (BH‐adjusted P ≥ 0.05). The dataset comprised nine unique participants and 16 recording sessions, yielding 48 tone‐level wet‐dry paired observations per feature.

All nine participants initially completed the recordings with CFPEs first and standard Ag/AgCl wet electrodes second. Seven of these participants returned for an additional session with the reverse order, in which wet electrodes were tested first and CFPEs second. The study therefore included nine unique participants and 16 recording sessions. The FFR waveforms (Figure 6b,c) show a typical structure consisting of a transient onset and a sustained wave that follows the pitch of the stimulating sound. The responses obtained with CFPEs showed waveform features similar to those recorded with wet electrodes. The pitch contours of both wet and dry electrode responses mirrored the differences in lexical tone between the three stimulating sounds, with no observable difference between the electrode types. Inter‐trial phase coherence time‐frequency representations (ITPC TFR) showed clear spectral lines at the speech fundamental frequency and its harmonics for both electrode types. The analysis compared 18 FFR/LLR features using two‐tailed paired t‐tests, with multiple comparisons addressed using the Benjamini‐Hochberg (BH) false discovery rate procedure. No significant difference in FFR SNR was detected between wet electrodes and CFPEs (5.03 ± 4.56 vs. 5.02 ± 4.82, respectively; BH‐adjusted P = 0.9620) (Figure 6e). In contrast, LLR SNR was higher with CFPEs than with wet electrodes (12.45 ± 6.60 vs. 8.67 ± 4.00, respectively; BH‐adjusted P = 0.0044) (Figure 6d). Four additional features‐Power LOW, Power HIGH, Pitch Strength, and FFR Peak Amplitude‐also showed higher values with CFPEs after correction (BH‐adjusted P = 0.0044, 0.0070, 0.0070, and 0.0121, respectively). Thus, five of the 18 features showed statistically significant differences. Feature distributions and detailed statistical results are presented in Figure S17 and Table S6. The comparable signal fidelity of CFPEs in FFR and LLR acquisition is attributed to the stable, low‐impedance interface maintained by the three‐dimensional monolithic conductive architecture. These results demonstrate that the CFPE platform can resolve microvolt‐level neural signatures with signal quality comparable to gold‐standard Ag/AgCl wet electrodes, extending dry‐electrode electrophysiology beyond routine biopotential monitoring and motivating larger‐scale studies with randomized electrode order across diverse populations and recording conditions. Furthermore, compared to recently reported state‐of‐the‐art flexible dry electrodes (Table S7), the CFPE framework itself provides conductive pathways and compressible support, while PEDOT:PSS coats and bridges the fibers, combining preparation‐free hairy‐scalp compatibility with repeated‐use durability, bridging a critical gap by enabling the gel‐free acquisition of microvolt‐level neural signatures. Given the relevance of FFR and LLR to neurodevelopmental research, the gel‐free, preparation‐free nature of CFPEs may facilitate future recordings in young children who have difficulty tolerating conventional electrode preparation. Avoiding gel application and scalp abrasion may reduce preparation demands, while the soft interface may also benefit individuals with limited tolerance for scalp preparation.

## 3 Conclusion

We have developed a carbon fiber‐based porous dry electrode (CFPE) with a three‐dimensional monolithic conductive architecture for gel‐free electrophysiological recording. The electrode is fabricated via carbonization of PAN fibers, electrochemical surface functionalization, and PEDOT:PSS coating, yielding a monolithic porous structure with volumetric ionic‐electronic conductivity. The CFPE achieves an electrode‐skin impedance comparable to wet electrodes without skin preparation and, critically, maintains stable impedance over 6 h of continuous wear while wet electrodes, applied with standard skin preparation, degrade by 56‐fold, thereby eliminating the gel‐dehydration failure mode that limits prolonged electrophysiological recording. The electrode withstands 10,000 compression cycles, 21 days of NaCl exposure, and 8 weeks of storage with minimal performance degradation. High‐fidelity ECG, EMG, and EEG recordings with >90% correlation coefficient and SNR comparable to wet Ag/AgCl electrodes were demonstrated. Additional ECG recordings showed identifiable R peaks during slow walking with both fresh and durability‐challenged CFPEs, although motion‐related waveform distortion remained. The acquisition of microvolt‐level FFR and LLR signals demonstrates the feasibility of gel‐free auditory‐evoked recording with CFPEs. The analysis showed higher LLR SNR with CFPEs after correction for multiple comparisons, while no significant difference in FFR SNR was detected. These results establish the CFPE as a viable platform for neurophysiology applications that currently require wet electrodes and may facilitate future translation to continuous monitoring scenarios and populations for whom gel‐based electrode preparation is impractical.

## 4 Materials and Methods

### 4.1 Materials

Polyacrylonitrile (PAN) fiber felt was supplied by Sichuan CHEMSHINE CARBON Co., LTD. PEDOT:PSS aqueous solution (PH1000) was purchased from Heraeus Co. All chemicals were used as received without further purification. Dimethyl sulfoxide (DMSO) was obtained from Shanghai Macklin Biochemical Technology Co., Ltd. Fluorocarbon surfactant (FC) (FC‐4430, >95%) was obtained from 3M Company. The deionized (DI) water used in this work was purified by Milli‐Q system, ≥18.2 MΩ·cm (Millipore, USA).

### 4.2 Carbon Fiber Synthesis

The PAN fiber felt was cleaned with acetone, rinsed with deionized water, and dried before use. It was placed at the center of a 2‐inch quartz tube furnace and heated in air at 230°C for 80 min. Argon was then supplied at 400 sccm, and the furnace temperature was increased to 950°C and maintained for 30 min to carbonize the fibers. Afterward, the sample was allowed to cool to room temperature under flowing argon.

### 4.3 Surface Modification of Carbon Fiber

Electrochemical treatment was carried out in a three‐electrode cell containing 1 M H_2_SO_4_. The carbon fiber cube was used as the working electrode, with a platinum sheet and an Ag/AgCl electrode serving as the counter and reference electrodes, respectively. A constant potential of 1.2 V was applied for 5 min in chronoamperometry mode using an electrochemical workstation. The treated carbon fibers were then rinsed thoroughly with deionized water.

### 4.4 Fabrication of CFPEs

Carbon fiber felt was punched into cylindrical samples with a diameter of 10 mm. The PEDOT:PSS coating solution was prepared by mixing 10 g of Clevios PH1000 dispersion with 0.5 g of DMSO and 0.1 g of FC, followed by stirring at room temperature for 1 h. The carbon fiber samples were immersed in this solution and placed under vacuum in a desiccator for 30 min to promote infiltration of the coating into the fiber network. The samples were then removed, dried at 90°C for 6 h, and annealed in a tube furnace at 140 °C for 1 h under flowing Ar (200 sccm). Unless otherwise specified, CFPEs prepared with one PEDOT:PSS coating cycle were used for subsequent characterization and performance evaluations.

### 4.5 Materials Characterization

Raman spectra were recorded using a WiTec Alpha 300 system with a 532 nm excitation laser before and after carbonization to examine the conversion of PAN fibers into carbon fibers. Surface chemical changes were analyzed by X‐ray photoelectron spectroscopy (XPS; K‐Alpha, Thermo Scientific). The surface morphology was observed by scanning electron microscopy (SEM; JEOL‐7800F) at an accelerating voltage of 5 kV.

### 4.6 Mechanical Properties Characterization

Compression tests were performed using an MTS QTest/2L eP2 testing machine equipped with a 225 lbf (1 kN) load cell. Uniaxial compression was applied at a displacement rate of 10 mm min^−1^ and the load cell was calibrated before testing. Cyclic compression tests were carried out using a custom‐built tensile‐compression system at 100 mm min^−1^. The electrical response during cycling was recorded using a Keithley DMM6500.

### 4.7 Ethics Approval and Participant Consent

Skin irritation and sensitization tests were conducted by Guangdong Neway Quality Technology Service Co., Ltd. (China), a CMA‐certified laboratory (Certificate No. 240015340124) accredited by CNAS (Registration No. L9783), under experimental animal use license No. SYXK (Guangdong) 2024‐0160. The animal experiments were approved under animal ethics approval No. IACUC‐24012359 and performed in accordance with GB/T 16886.23‐2023 and GB/T 16886.10‐2024 for skin irritation and sensitization, respectively. The auditory‐evoked EEG experiments, including FFR and LLR recordings and the associated auxiliary physiological recordings and electrode‐contact impedance checks specified in the approved protocol, were approved by the Joint Chinese University of Hong Kong‐New Territories East Cluster Clinical Research Ethics Committee (CREC Ref. No. 2021.485). Written informed consent was obtained from these participants before participation.

### 4.8 Contact Impedance Measurement

The electrode‐skin contact impedance measurements were conducted under laboratory ambient conditions (approximately 22 °C, 60% relative humidity), with no procedure used to accelerate gel drying. The same custom fixation strap was used for the CFPE and for the commercial wet electrode, applying a contact pressure in the range 3.01 kPa to 7.25 kPa throughout the test. For measurements performed with skin preparation, the measurement site was gently cleaned and prepared using NuPrep. For measurements without skin preparation, no abrasive paste or other skin‐preparation agent was applied. Conductive gel was used only for the wet electrodes. In the 6 h continuous‐wear test the commercial wet electrode was applied following standard practice for wet electrodes, with skin preparation and with fresh conductive gel (Ten 20) applied immediately before the start of the test and not replenished thereafter, whereas the CFPE was applied without skin preparation and without gel. The CFPEs were placed proximally to the wrist, while two commercial wet electrodes were positioned at the mid‐forearm and proximal to the elbow, serving as the reference electrode and counter electrode, respectively. Electrode‐skin contact‐impedance spectra were measured using a PalmSens MultiPalmSens 4 potentiostat. Routine measurements were performed over 10 Hz‐10 kHz, whereas spectra used for equivalent‐circuit fitting and durability evaluations were acquired over 1 Hz‐100 kHz.

### 4.9 Biocompatibility Test

CFPE extracts were prepared by adding a known mass of CFPEs to complete culture medium at 100 mg mL^−^^1^ and incubating the mixture at 4°C for 24 h. The extract was sterilized by filtration before use. L929 cells in the exponential growth phase were seeded in 96‐well plates at 5,000 cells per well for the CCK‐8 assay and in confocal dishes at 50,000 cells per dish for Live/Dead staining. After cell attachment, 100 µL of CFPE extract was added to each well of the 96‐well plates, while 1 mL was added to each confocal dish. The cells were incubated with the extracts for 24 or 48 h.

For the CCK‐8 assay, 10 µL of CCK‐8 reagent was added to each well, followed by incubation at 37°C in 5% CO_2_ for 2 h. Absorbance was measured at 450 nm using a microplate reader (Tecan, Switzerland). Cell viability was calculated relative to the blank control.

For Live/Dead staining, the cells were washed twice with PBS and incubated with 100 µL of staining solution containing 2 µM Calcein‐AM and 4 µM propidium iodide for 30 min at 37°C in the dark. The cells were then washed twice with PBS before imaging by confocal laser scanning microscopy (Leica TCS SP8, Germany). Calcein‐AM fluorescence was detected at 515 nm with 488 nm excitation, while propidium iodide fluorescence was detected at 617 nm with 535 nm excitation.

### 4.10 Electrophysiological Signal Recording

To compare signal quality during physiological activity electrical recordings, commercial wet electrodes and CFPEs were employed simultaneously. ECG, EMG, and resting‐state EEG were recorded using an OpenBCI Cyton board on the first author. Collected data underwent fundamental signal analysis (including filtering, spectrogram generation, and Fast‐Fourier Transform) using MATLAB, with dedicated signal processing algorithms applied for analysis. The Pearson correlation coefficient between signals recorded with CFPEs and commercial Ag/AgCl electrodes was calculated as:

$$R\;=\frac{\mathrm{cov}\left(x1,x2\right)}{\sqrt{\mathrm{cov}\left(x1,x1\right)\mathrm{cov}\left(x2,x2\right)}}$$

where x_1_ and x_2_ are the filtered signals recorded using the Ag/AgCl electrode and CFPE, respectively, and cov(x_i_, x_j_) denotes the covariance between the two signals.

Dynamic ECG signals were recorded during seated rest and slow walking at 3.6 km h^−^^1^. Fresh wet electrodes, fresh CFPEs, commercial dry electrodes, CFPEs after 8,000 compression cycles, CFPEs after 100 application/removal cycles, and CFPEs after 50 artificial‐sweat cycles at pH 4.7 were evaluated simultaneously. The six test electrodes were positioned in proximity on the left forearm and connected to six independent recording channels of an OpenBCI Cyton board, enabling simultaneous comparison under identical physiological and motion conditions. A common reference electrode was placed on the right forearm, forming a Lead I‐like ECG recording configuration, while the OpenBCI BIAS/ground electrode was placed on the lower right leg. Signals were acquired at a sampling rate of 1000 Hz using an OpenBCI Cyton board equipped with a WiFi Shield and recorded on a computer using the OpenBCI GUI. For visualization of ECG waveforms, the recordings were processed using a 0.5‐40 Hz band‐pass filter and a 50 Hz notch filter. To descriptively assess low‐frequency motion‐related fluctuations, the ECG signals were separately filtered between 0.05 and 0.5 Hz, and the RMS value was calculated for each electrode condition. Measurements were performed in three experimental runs in the same participant, using a different electrode specimen for each condition in each run. RMS values are reported as mean ± SD across the three runs for descriptive comparison. These measurements reflect variation across electrode specimens and recording runs, rather than between participants.

### 4.11 Auditory‐Evoked EEG Experiment

All nine participants completed an initial session with CFPE recordings followed by standard Ag/AgCl wet‐electrode recordings. Seven participants subsequently completed an additional session with wet electrodes first and CFPEs second. Skin preparation using NuPrep was performed before wet‐electrode placement. In the wet‐first sessions, residual conductive gel was removed using wet wipes, and the skin was wiped dry before CFPEs were applied at the same recording sites. Signals were recorded without online filtering and exported to MATLAB for offline analysis. A notch filter was applied to remove 50 Hz line noise and its harmonics. For FFR analysis, the Cz signal was re‐referenced to the average of the left and right mastoid signals, filtered using an 80–1500 Hz FIR band‐pass filter, and segmented into epochs from ‐50 to 270 ms relative to stimulus onset. For LLR analysis, the data were filtered between 0.1 and 40 Hz and segmented from ‐100 to 500 ms relative to stimulus onset. Details of the feature‐extraction procedures have been described in our previous studies [31, 32].

### 4.12 Statistical Analysis

FFR/LLR analyses included nine unique participants, seven of whom completed an additional reverse‐order session. Each session included three stimulus tones. In the reported analysis, each tone‐specific wet‐dry comparison was treated as a separate paired observation, yielding 48 paired observations per feature (df = 47). No additional transformation was applied to the extracted features before statistical analysis, and no observations were excluded as outliers. Descriptive data are presented as mean ± SD. Electrode‐related differences were assessed using two‐tailed paired t‐tests. Multiple comparisons across the 18 FFR/LLR features were addressed using the Benjamini‐Hochberg (BH) false discovery rate procedure, with statistical significance defined as a BH‐adjusted P value < 0.05. Unadjusted and BH‐adjusted P values are reported in Table S6. Statistical analyses were performed using MATLAB and Python.

## Author Contributions

**Haoxuan Sun**: conceptualization, methodology, data curation, project administration, visualization, validation, Writing – original draft. **Nikolay Novitskiy**: data curation, visualization, writing – original draft, formal analysis, methodology. **Dongwook Jang**: conceptualization, data curation, formal analysis, methodology. **Yixiang Liu**: visualization, data curation. **Shaoqi Pan**: conceptualization, data curation, visualization. **Ting Huang**: conceptualization, formal analysis. **Yitong Li**: conceptualization, data curation, validation. **Huisi Yang**: conceptualization, formal analysis. **Leonardo Nicholas Adi Wijaya**: conceptualization, methodology. **Md Masruck Alam**: conceptualization, validation. **Patrick Cm wong**: methodology, supervision, writing – review and editing, funding acquisition, resources. **Zhaoli Gao**: conceptualization, writing – review and editing, supervision, project administration, resources, funding acquisition.

## Conflicts of Interest

Z. Gao, P. Wong, H. Sun, N. Novitskiy, and S. Pan are named inventors on a patent application related to this work, submitted by The Chinese University of Hong Kong (Application No. 64/146173). The remaining authors declare no competing interests.

## Supporting information




**Supporting File**: advs78531‐sup‐0001‐SuppMat.pdf.

## Data Availability

The data that support the findings of this study are available from the corresponding authors upon reasonable request.
