# Supplementary material for: Gel‐Free Capture of Microvolt‐Level Neural Signatures Via a 3D Monolithic Carbon Fiber Dry Electrode
Source: Adv Sci (Weinh). 2026 Oct 29:e78531. Online ahead of print. doi: 10.1002/advs.78531 (PMC13720261; doi:10.1002/advs.78531)
Supplement: Supplementary file 1 [file ADVS-9999-e78531-s001.pdf]

Supporting Information

Gel-Free Capture of Microvolt-Level Neural Signatures via a 3D Monolithic Carbon Fiber Dry Electrode

Haoxuan SUN, Nikolay NOVITSKIY, Dongwook JANG, Yixiang LIU, Shaoqi PAN, Ting HUANG, Yitong LI, Huisi YANG, Leonardo Nicholas Adi WIJAYA, Md Masruck ALAM, Patrick CM WONG, Zhaoli GAO**

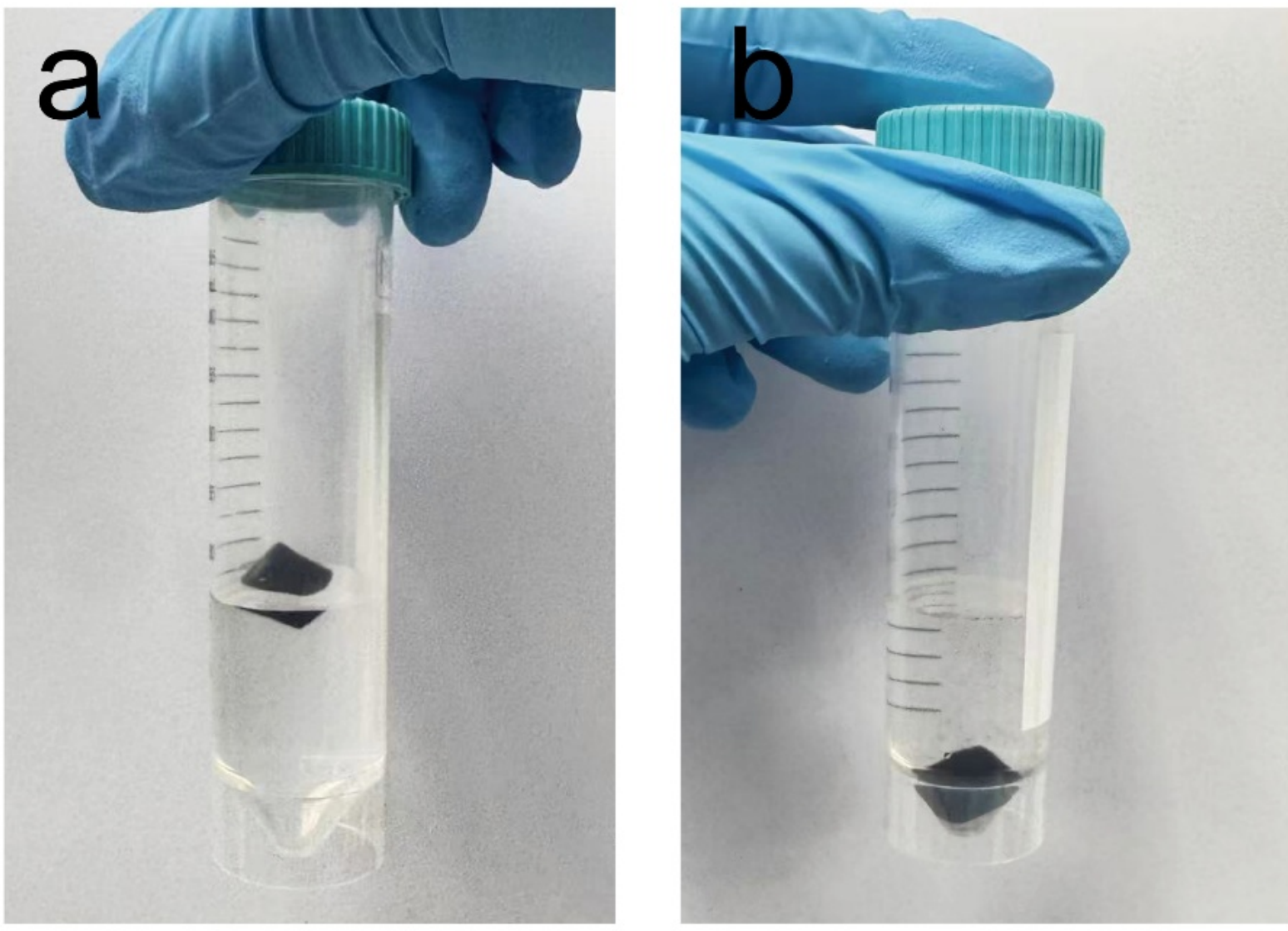

Figure S1. Carbon fiber cubes (a) before and (b) after modification were placed in centrifuge tubes containing deionized water. The unmodified carbon fiber cubes were hydrophobic and floated on the surface of the deionized water, while the modified ones transformed into hydrophilic and sank to the bottom of the deionized water.

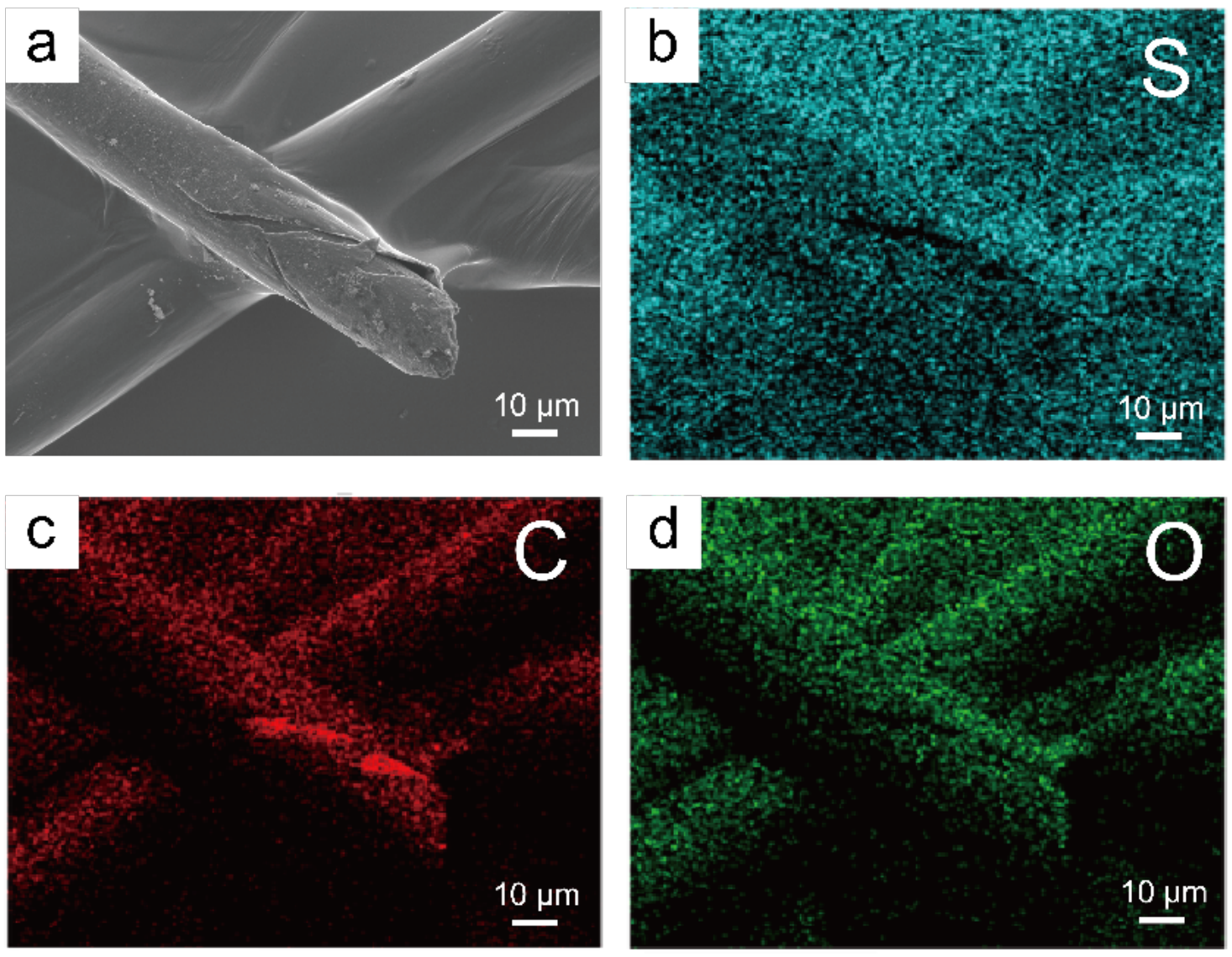

Figure S2. (a) Enlarged SEM image of PEDOT: PSS-coated carbon fiber and the corresponding EDX elemental mapping patterns of (b) S, (c) C, and (d) O. A coating crack is observed in the middle of the image. Within this crack region, an intense distribution of C element is clearly visualized, while the intensities of S and O elements almost vanish compared to other areas, confirming that the PEDOT:PSS film was successfully coated on the carbon fiber surface.

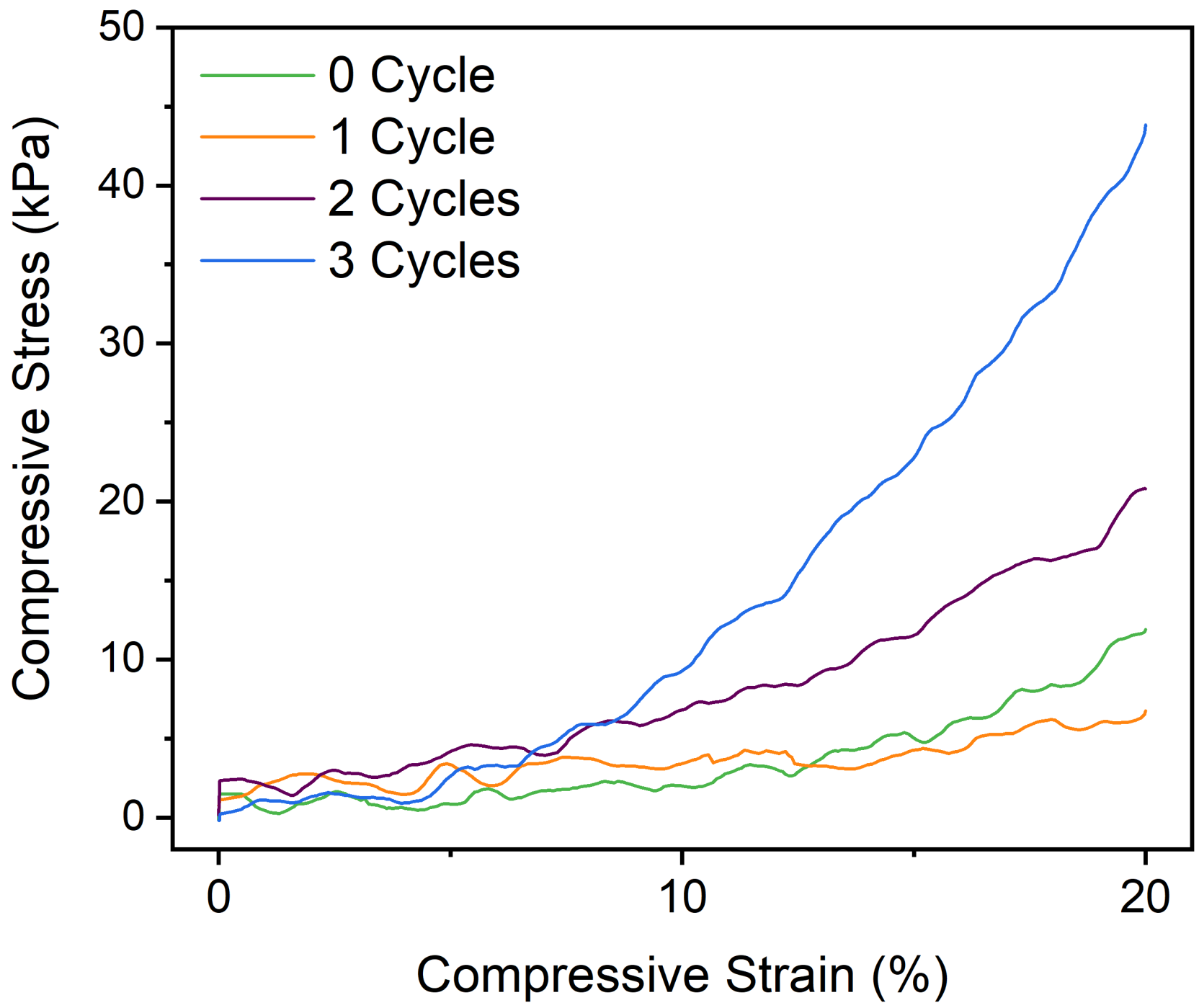

Figure S3. Stress-strain curves of CFPE with different immersion cycles in the compressive strain range of 0-20% at a maximum compressive strain of 70%.

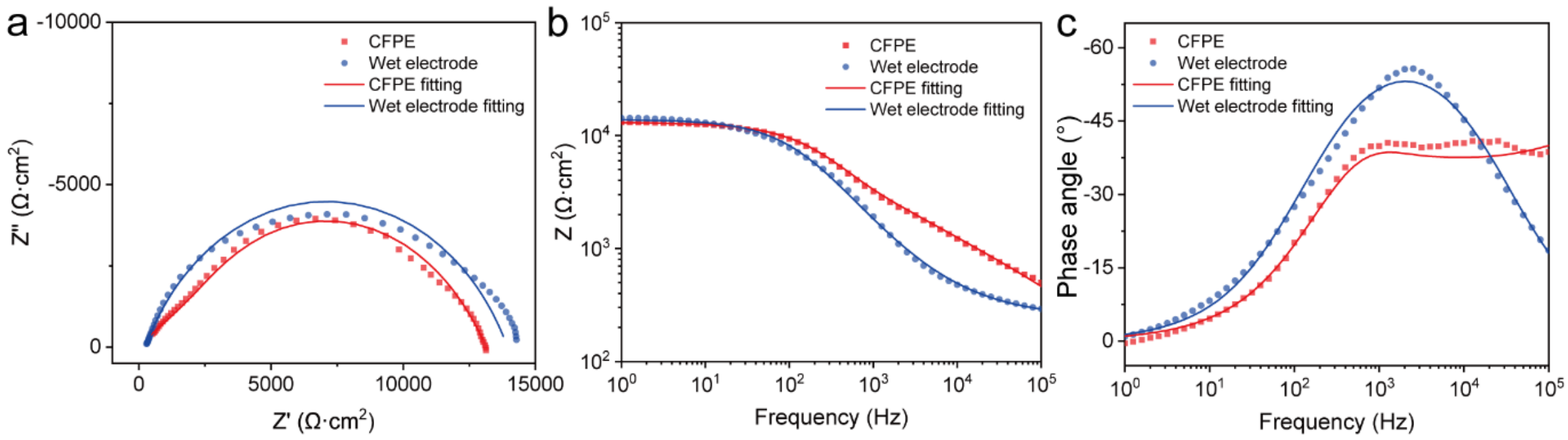

Figure S4. Equivalent-circuit fitting of the electrode-skin impedance spectra for the wet electrode and CFPE. (a) Nyquist plots, (b) impedance-magnitude plots, and (c) phase-angle plots of the experimental and fitted spectra. Symbols represent the experimental data, and solid lines represent the corresponding ZView fitting curves. R1 represents the lumped ohmic resistance arising from the electrode, lead, and ionic/skin conduction pathway, R2 represents the effective interfacial charge-transfer and polarization resistance. CPE denotes a constant phase element describing the non-ideal capacitive response of the heterogeneous electrode-skin interface, and W denotes a Warburg element representing a diffusion-related impedance contribution.

The measured impedance spectra were fitted using R1 + (CPE || R2) for the wet electrode and R1 + [CPE || (R2 + W)] for CFPE (Figure S4). R1 represents the lumped ohmic resistance, R2 describes an effective interfacial resistive contribution, and the constant phase element (CPE) accounts for non-ideal capacitive behavior.

For the wet electrode, the conductive gel provides a relatively continuous ionic pathway. Its measured response was described without a separate diffusion element; this does not imply the complete absence of diffusion-related processes. For CFPE, an additional Warburg element was included to represent a diffusion-related contribution, consistent with ionic transport within a heterogeneous, moisture-containing contact interface. This interpretation is phenomenological and does not independently establish the microscopic transport pathway.

The fitted R1 values were 48.9 and 316.6 $\Omega\cdot cm^2$ for CFPE and the wet electrode, respectively, while the corresponding R2 values were 1,337 and 17,435 $\Omega\cdot cm^2$ (Table S2). Because R2 forms part of different circuit configurations, these values should not be directly interpreted as a quantitative comparison of an identical physical charge-transfer resistance. No separate air-gap element was resolved; the possible effects of incomplete contact are incorporated into the effective interfacial response.

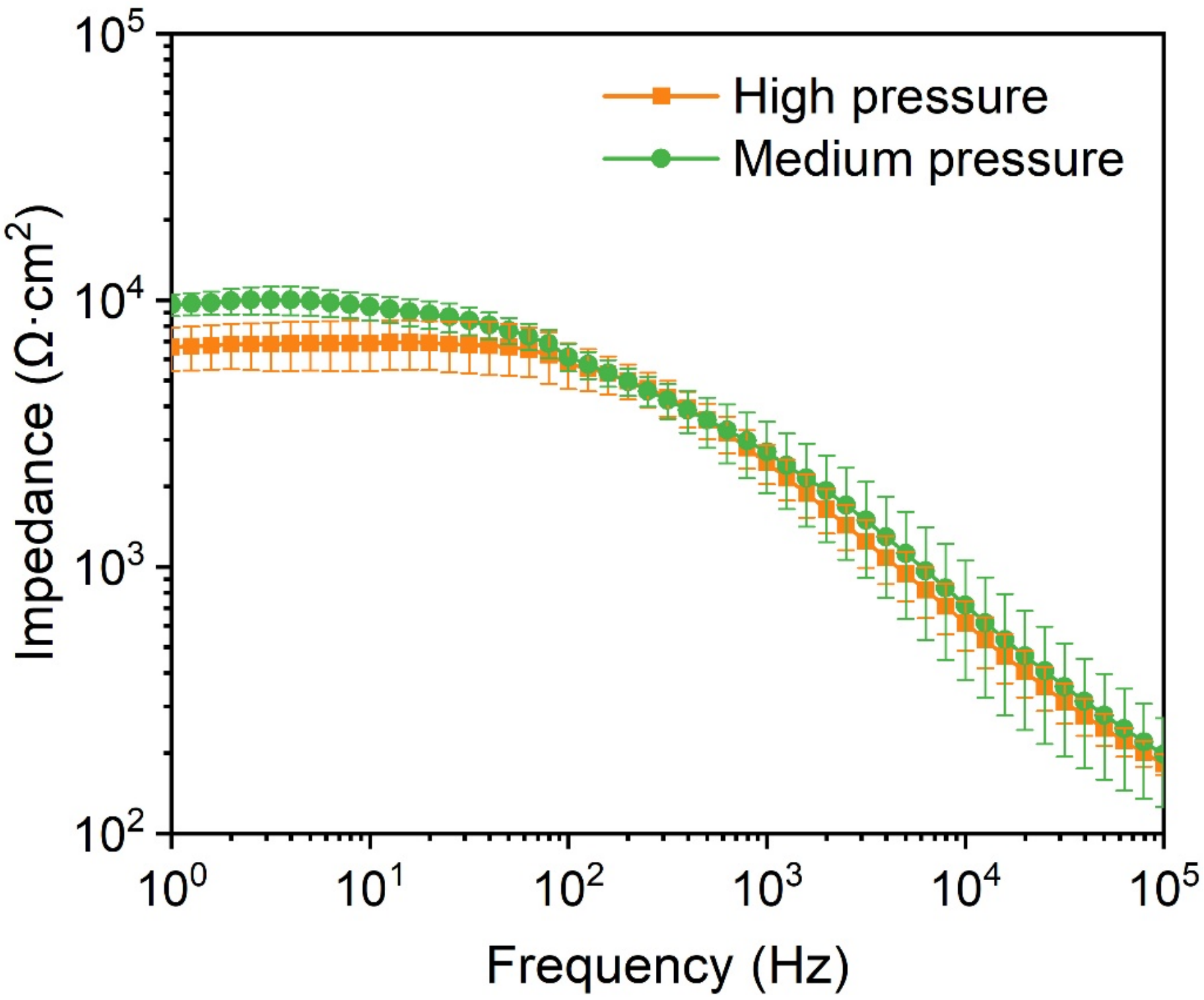

Figure S5. Pressure-dependent electrode-skin contact impedance of CFPE.

Under the routine strap-assisted contact condition of 3.01-7.25 kPa, fresh CFPEs exhibited a 10 Hz contact impedance of 11.69 ± 0.70 kΩ·cm² (Table S3). In a separate controlled loading experiment, the impedance was 9.4 kΩ·cm² at 18.27-22.05 kPa and decreased to 6.9 kΩ·cm² at 42.72-50.43 kPa, corresponding to an approximately 27% reduction. The routine measurement provides contextual reference rather than a matched baseline for the controlled loading experiment. The pressure-dependent reduction is consistent with improved effective contact and interfacial coupling under compression, but does not isolate the effect of porosity from other pressure-dependent contact effects.

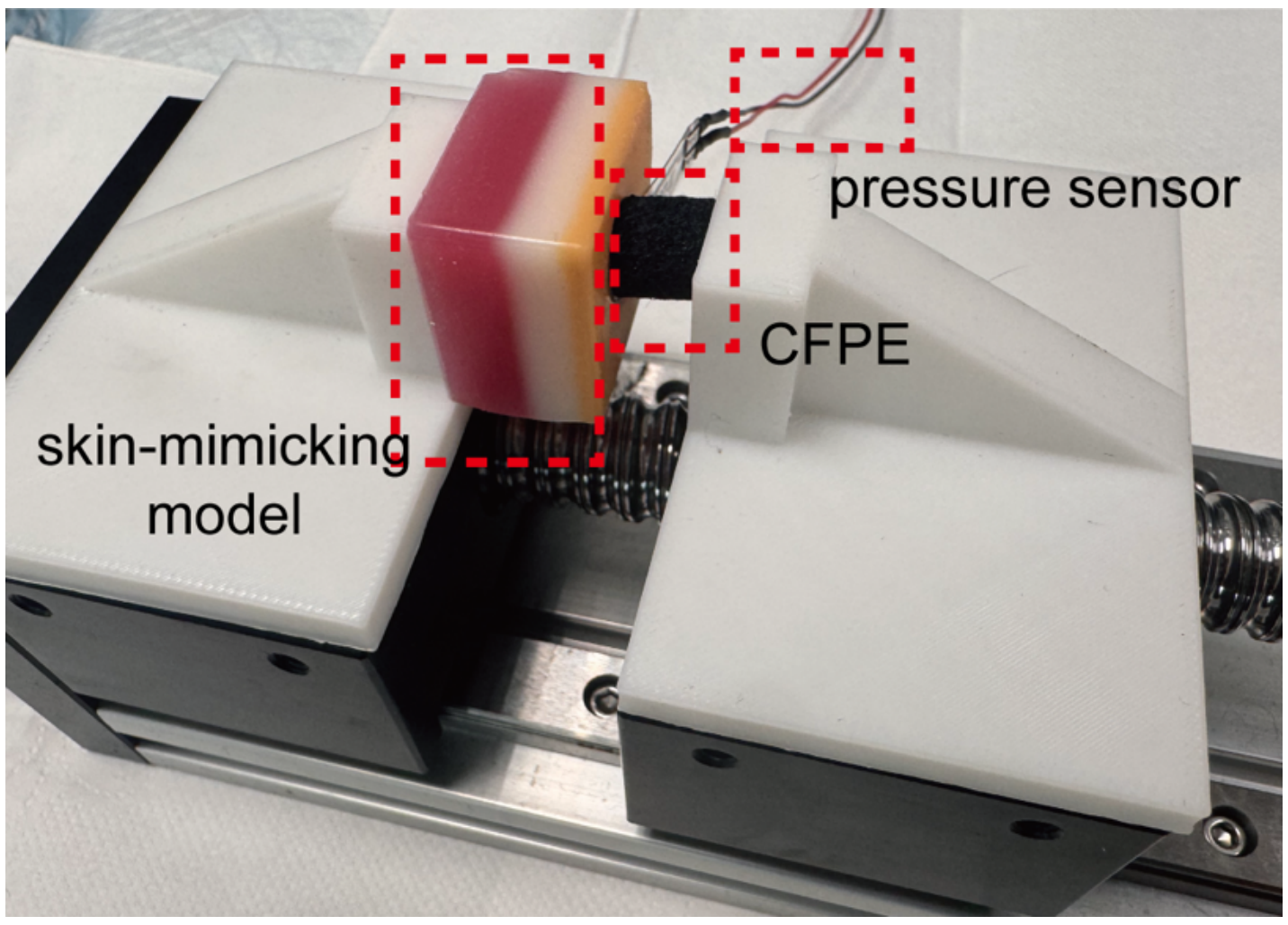

Figure S6. Experimental setup for cyclic compression of CFPEs on a skin-mimicking model. The CFPE was positioned between a commercial skin-mimicking model and the compression fixture. A pressure sensor was incorporated to monitor the applied contact pressure during the cyclic compression test. The setup was used to subject CFPEs to 8,000 compression cycles before contact-impedance measurement and post-challenge SEM characterization.

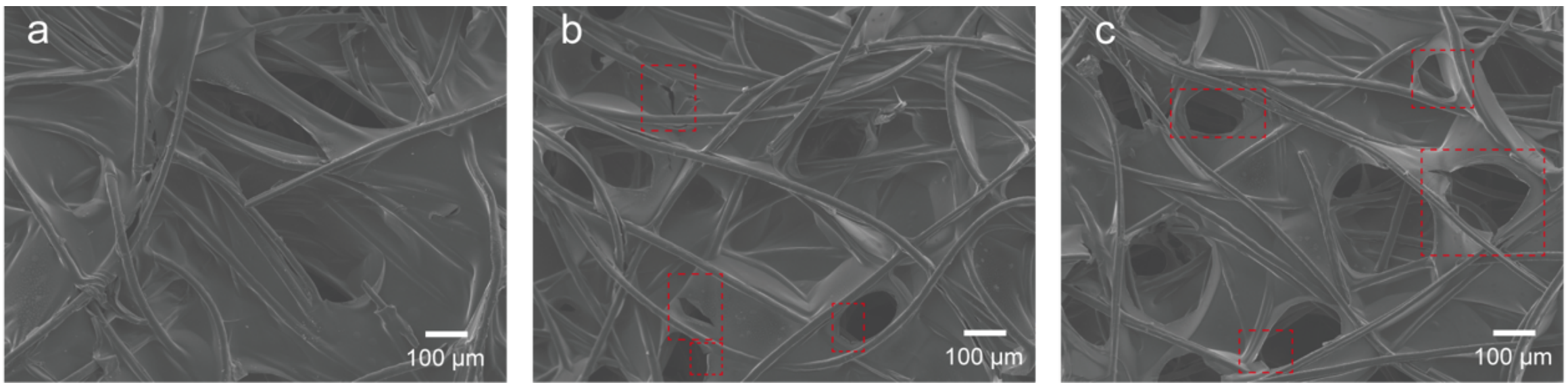

Figure S7. SEM characterization of CFPE surface morphology after cyclic compression. (a) Freshly prepared CFPE. (b) CFPE after 4,000 compression cycles. (c) CFPE after 8,000 compression cycles. Red dashed boxes highlight localized cracking and discontinuities in the PEDOT:PSS surface coating, which became more apparent with increasing compression cycles. The overall carbon-fiber network remained intact after the 8,000-cycle compression challenge. Scale bars: 100 µm.

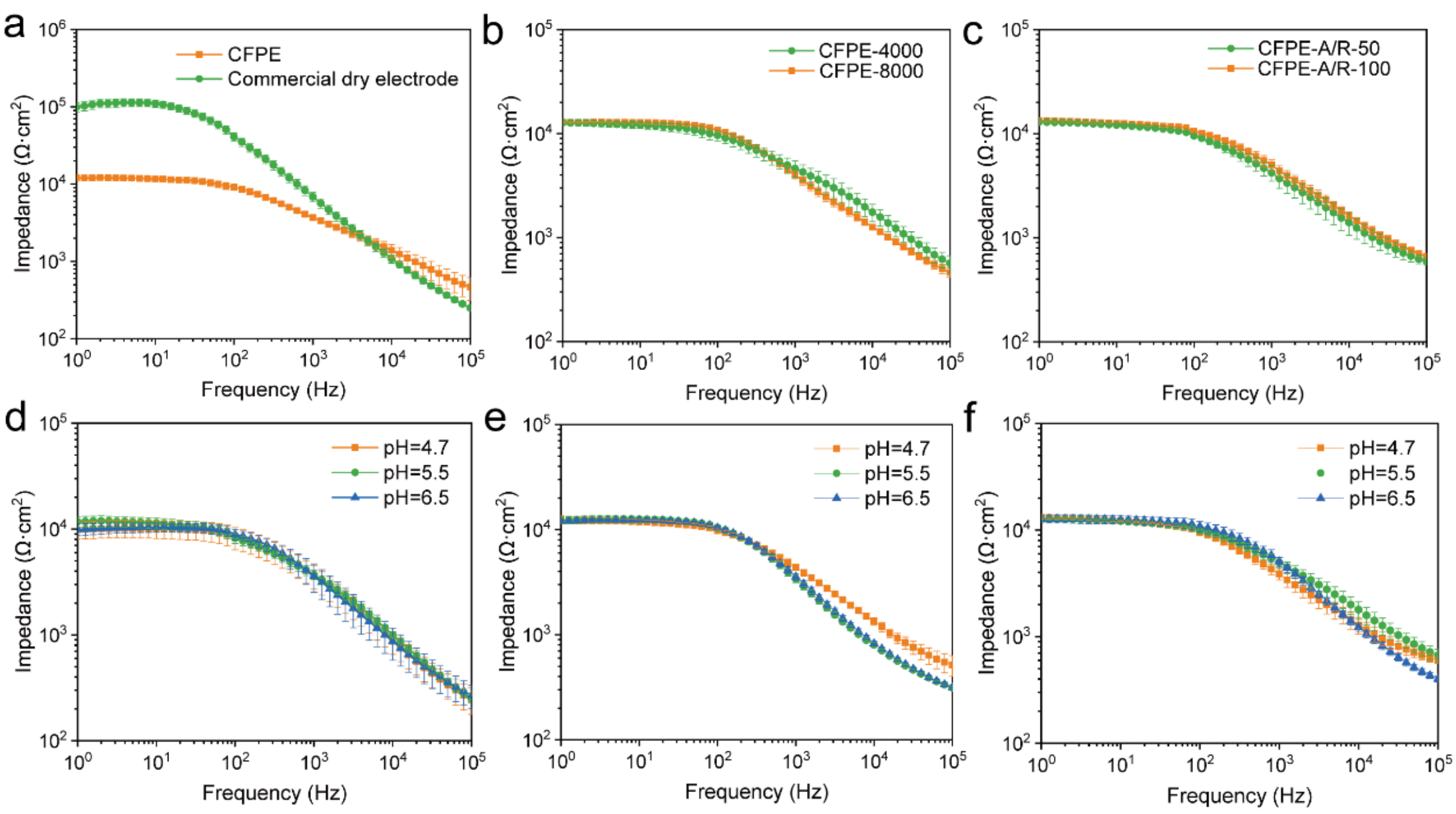

Figure S8. Frequency-dependent electrode-skin contact impedance of CFPEs after mechanical, repeated-use, and artificial-sweat cycling challenges. Contact-impedance spectra were measured from 1 Hz to 100 kHz. (a) Fresh CFPE and a commercial dry electrode (n = 6 electrodes). (b) CFPEs after 4,000 and 8,000 cyclic compression cycles on a skin-mimicking phantom (n = 3 electrodes). (c) CFPEs after 50 and 100 repeated application/removal cycles (n = 3 electrodes). (d-f) CFPEs after 1, 25, and 50 artificial-sweat wet-dry cycles, respectively, each panel compares pH 4.7, 5.5, and 6.5 (n = 3 electrodes). Data are presented as mean ± SD.

Cyclic-compression testing. CFPEs were positioned between a commercial skin-mimicking model and a compression fixture, with a pressure sensor monitoring the applied contact pressure (Figure S6). The electrodes underwent 8,000 compression cycles, and contact-impedance spectra were measured before cycling and after 4,000 and 8,000 cycles. The 10 Hz contact impedance was 11.69 ± 0.70 kΩ·cm² for fresh CFPEs and 12.13 ± 1.20 and 12.77 ± 0.29 kΩ·cm² after 4,000 and 8,000 cycles, respectively (Figure S8b and Table S3).

Repeated application/removal testing. CFPEs were repeatedly applied and removed using the same fixation configuration as that used for contact-impedance measurements. Each cycle consisted of a 1 min application period followed by removal and reapplication, for a total of 100 cycles. The 10 Hz contact impedance was 12.11 ± 0.28 and 12.56 ± 0.22 kΩ·cm² after 50 and 100 cycles, respectively, compared with 11.69 ± 0.70 kΩ·cm² for fresh CFPEs (Figure S8c and Table S3). As CFPE is a replaceable, cap- or strap-assisted electrode module, this test assessed repeated-use contact stability rather than adhesive strength.

Artificial-sweat cycling at different pH values. CFPEs were immersed in artificial sweat at pH 4.7, 5.5, or 6.5 for 30 min per cycle. After each immersion, excess liquid was gently blotted off, and the electrodes were dried at 40 °C and equilibrated at room temperature before further cycling or measurement. Contact-impedance spectra were acquired from 1 Hz to 100 kHz after 1, 25, and 50 cycles (Figure S8d-f). Across these measurement points and pH conditions, the mean 10 Hz contact impedance ranged from 10.07 to 12.53 kΩ·cm² (Table S3).

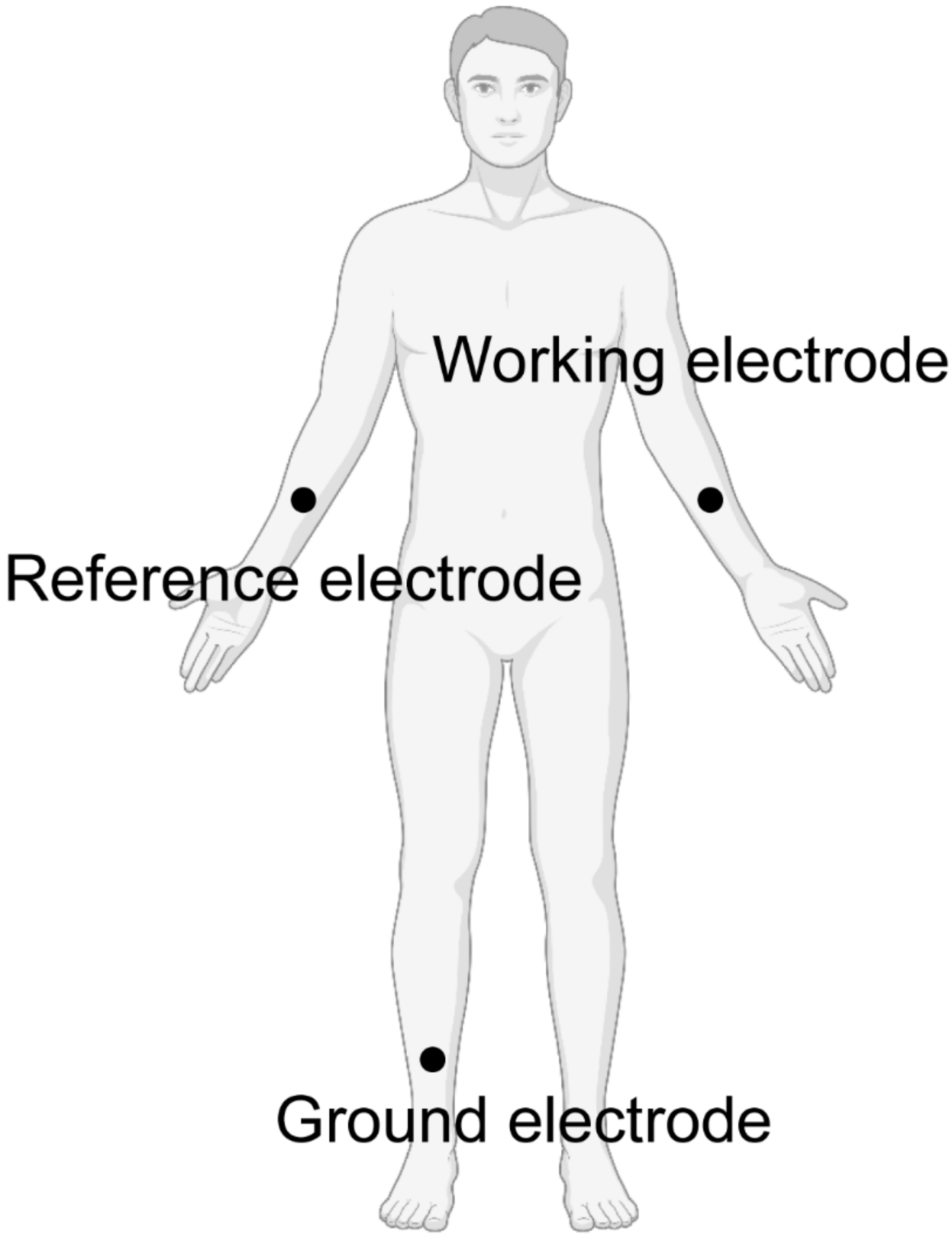

Figure S9. Schematic diagram of ECG recording setup.

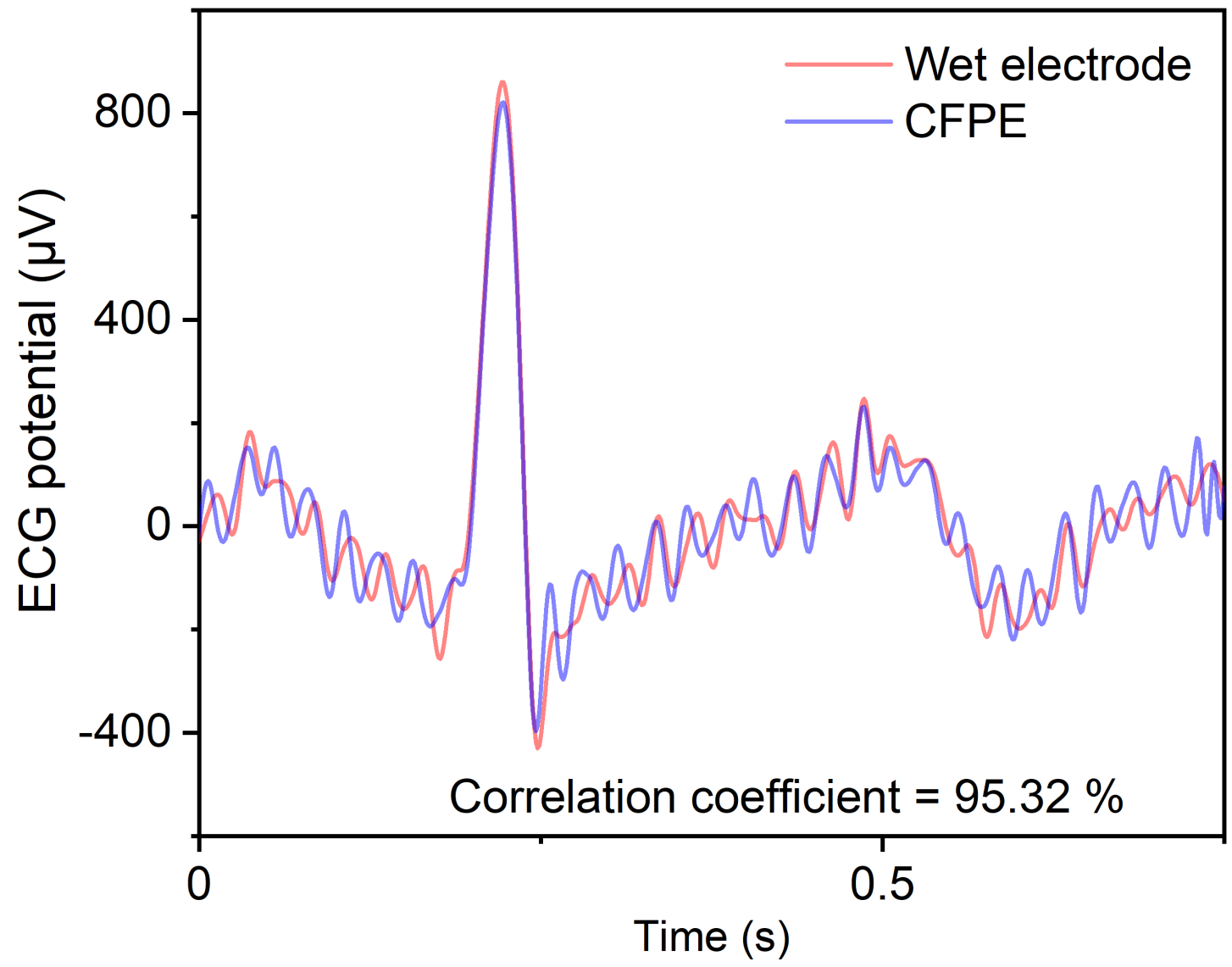

Figure S10. Comparison of ECG signal waveforms recorded simultaneously by both electrodes.

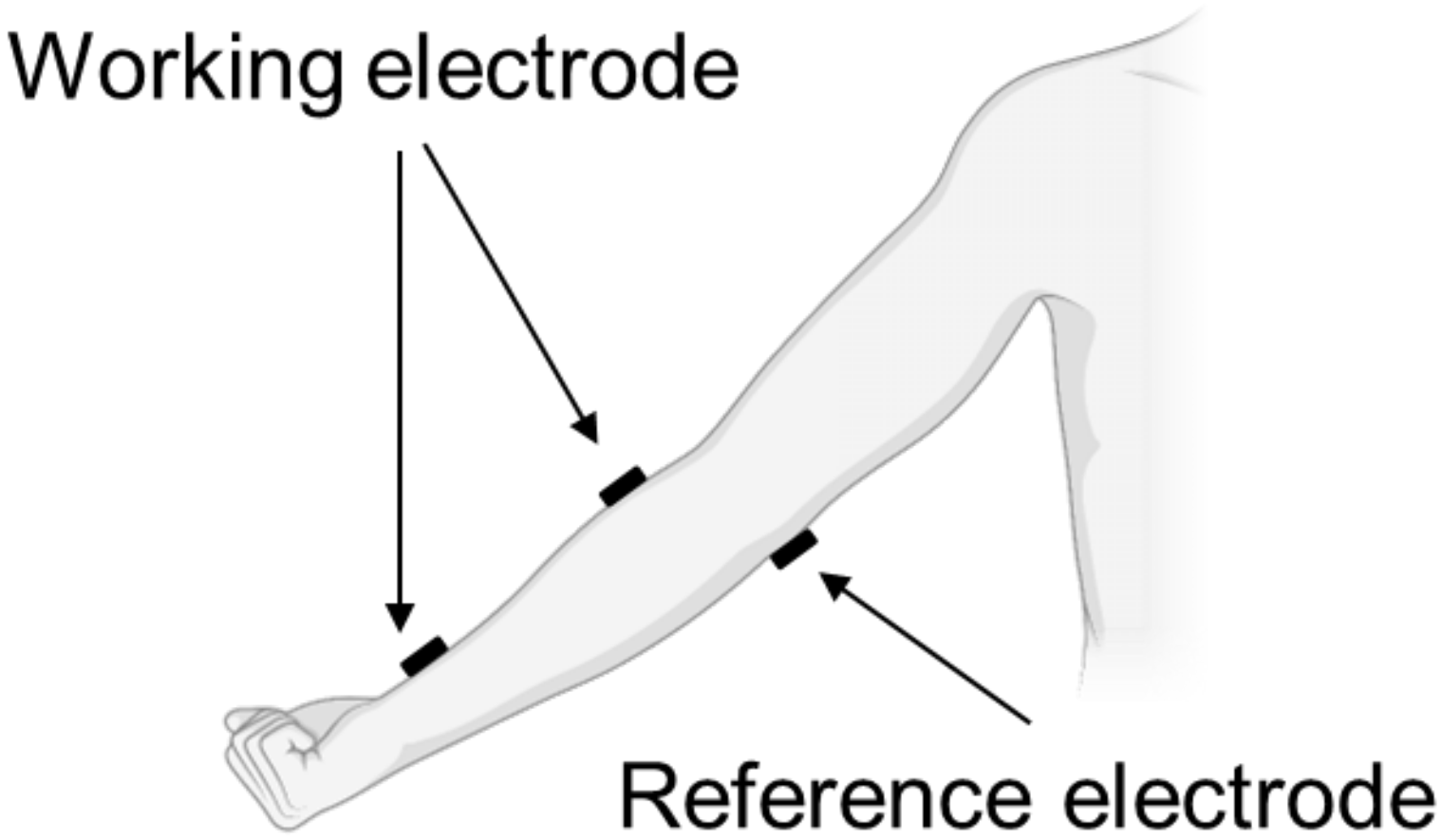

Figure S11. Schematic diagram of EMG recording setup.

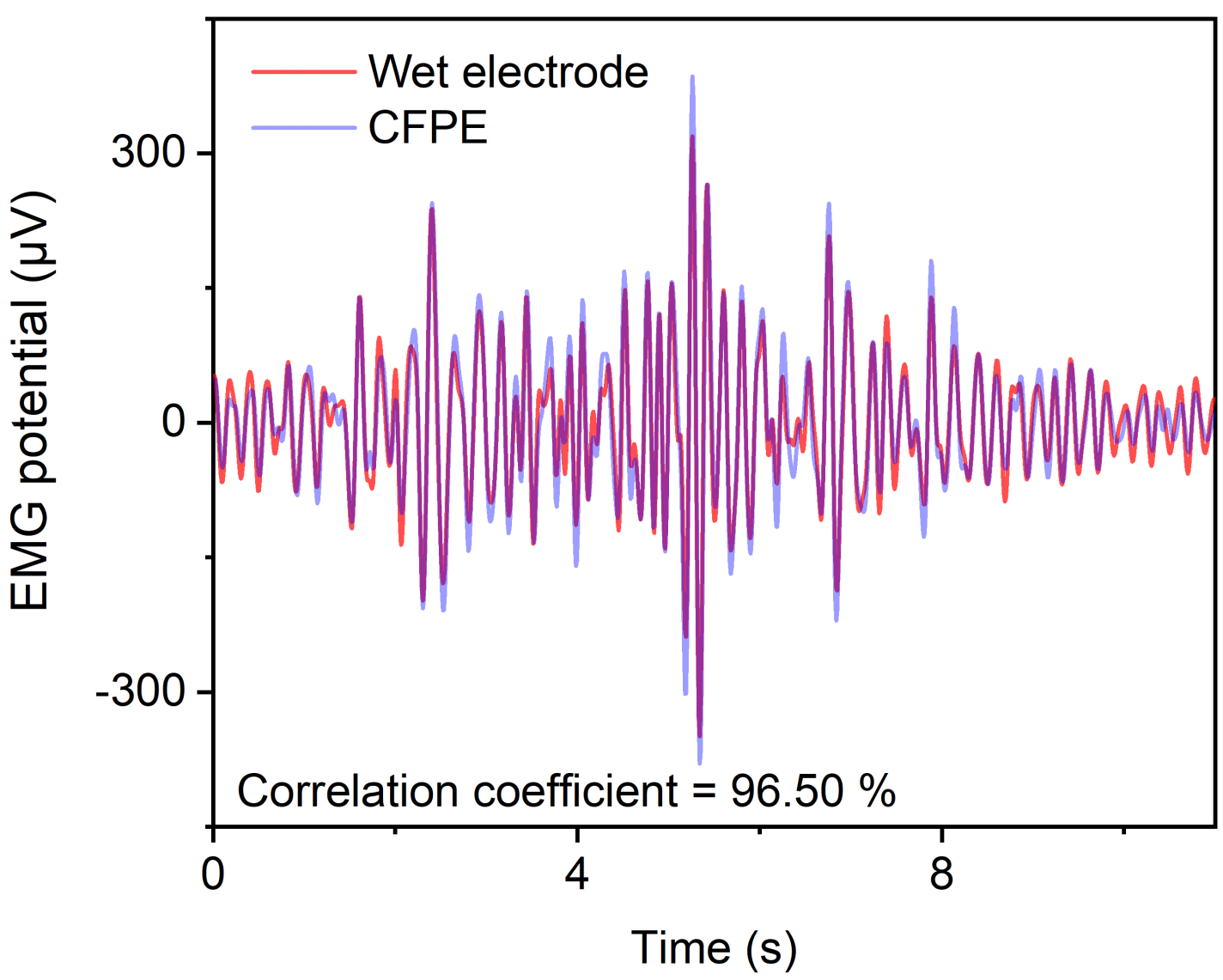

Figure S12. Comparison of EMG signal waveforms recorded simultaneously by both electrodes.

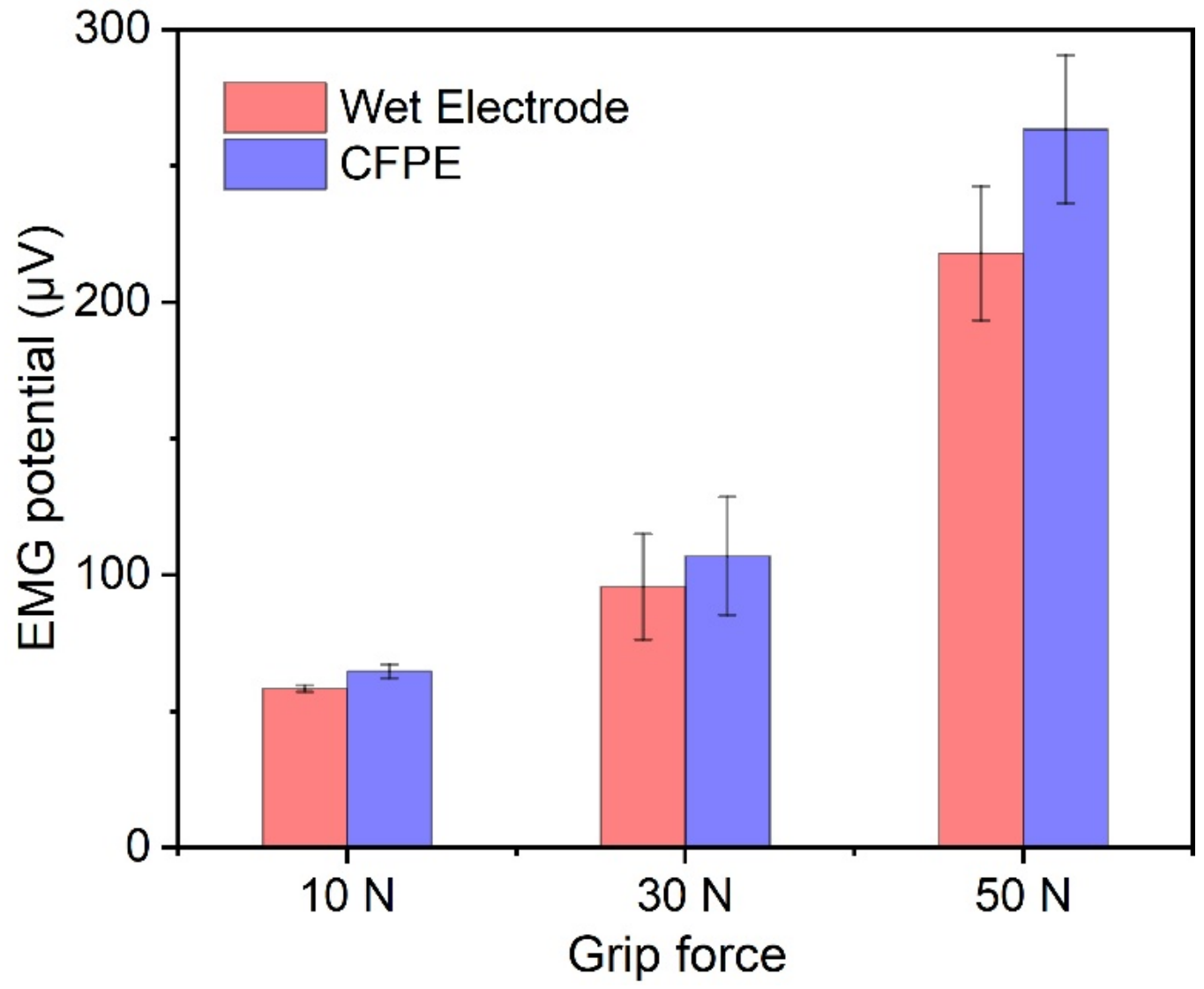

Figure S13. EMG signal amplitude at different grip-force levels. Data are presented as mean ± SD from three electrode specimens per electrode type (n = 3).

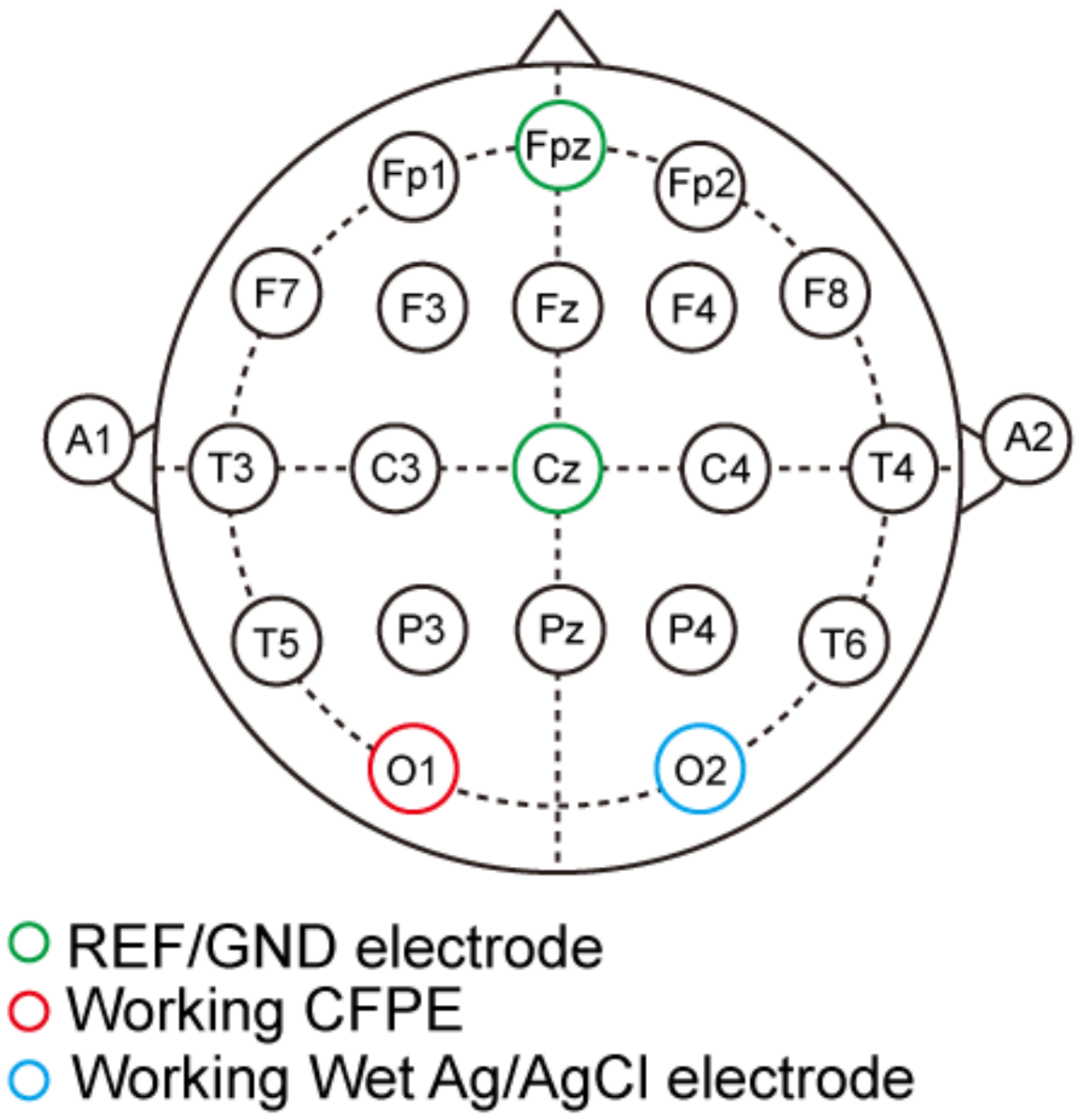

Figure S14. Placement of electrodes on the scalp in the eye closure and eye-opening test.

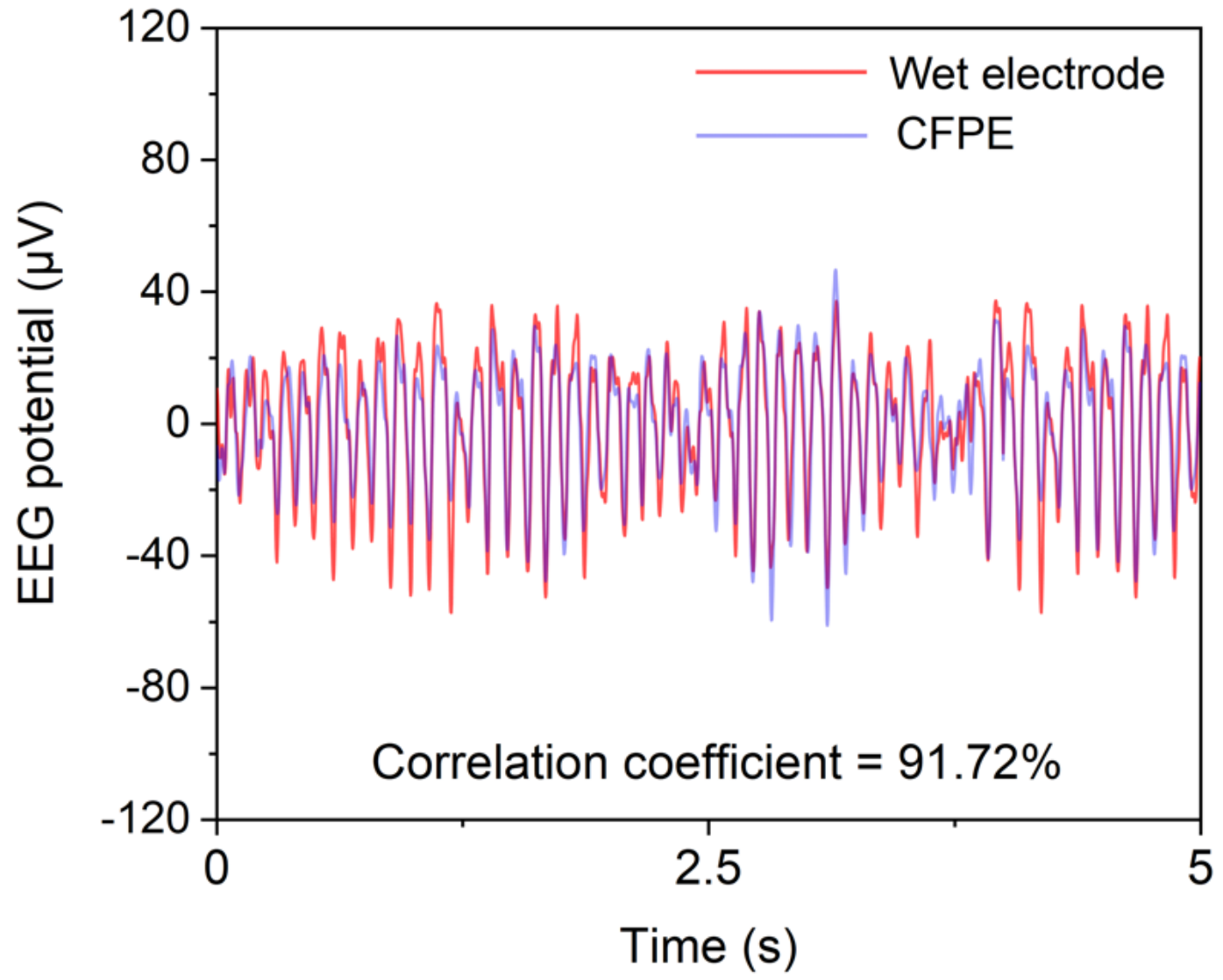

Figure S15. Comparison of EEG signal waveforms recorded simultaneously by both electrodes.

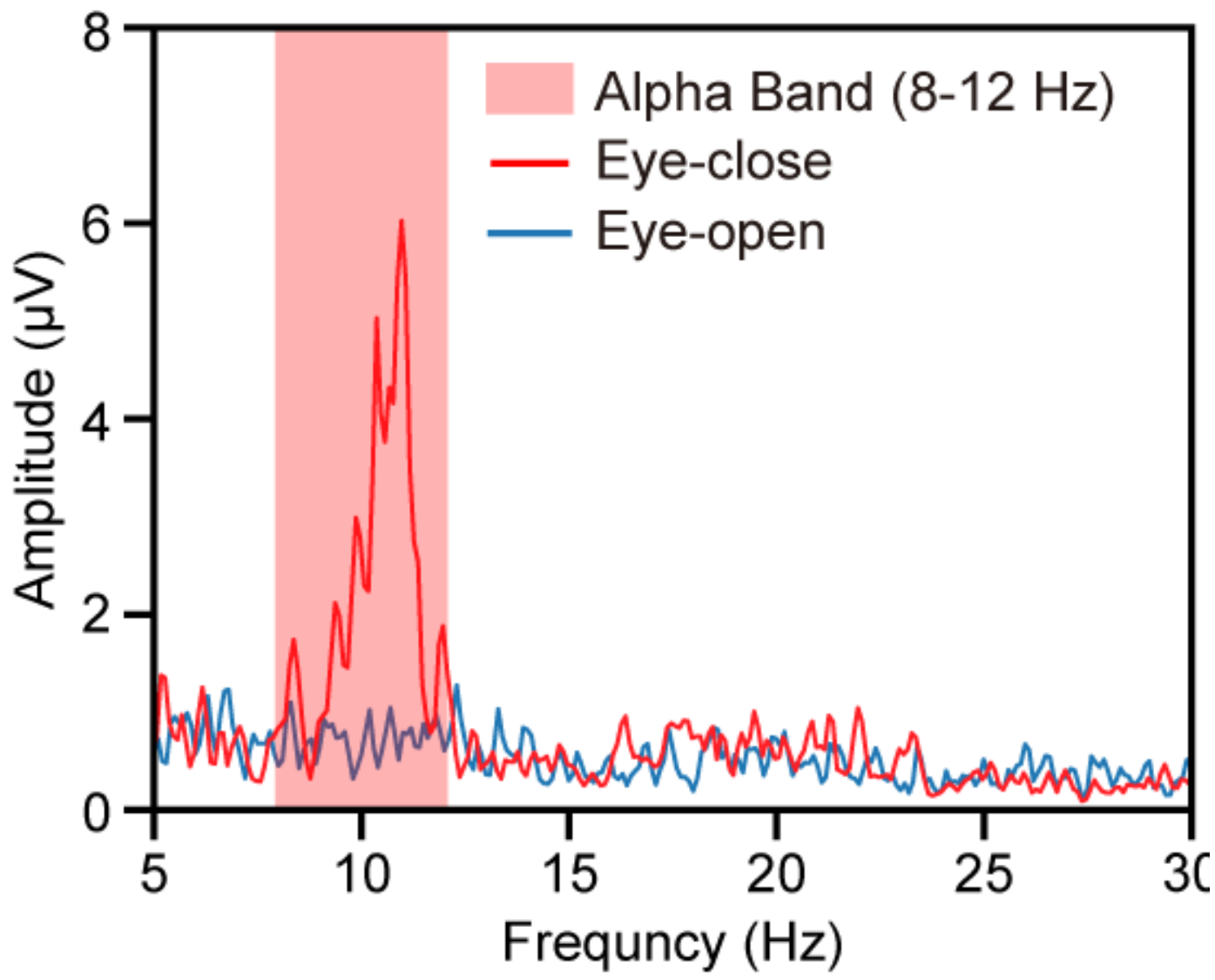

Figure S16. EEG power spectral density (PSD) under the eyes-closed (red) and eyes-open (blue) conditions, showing an evident alpha-band power increase during eyes closure.

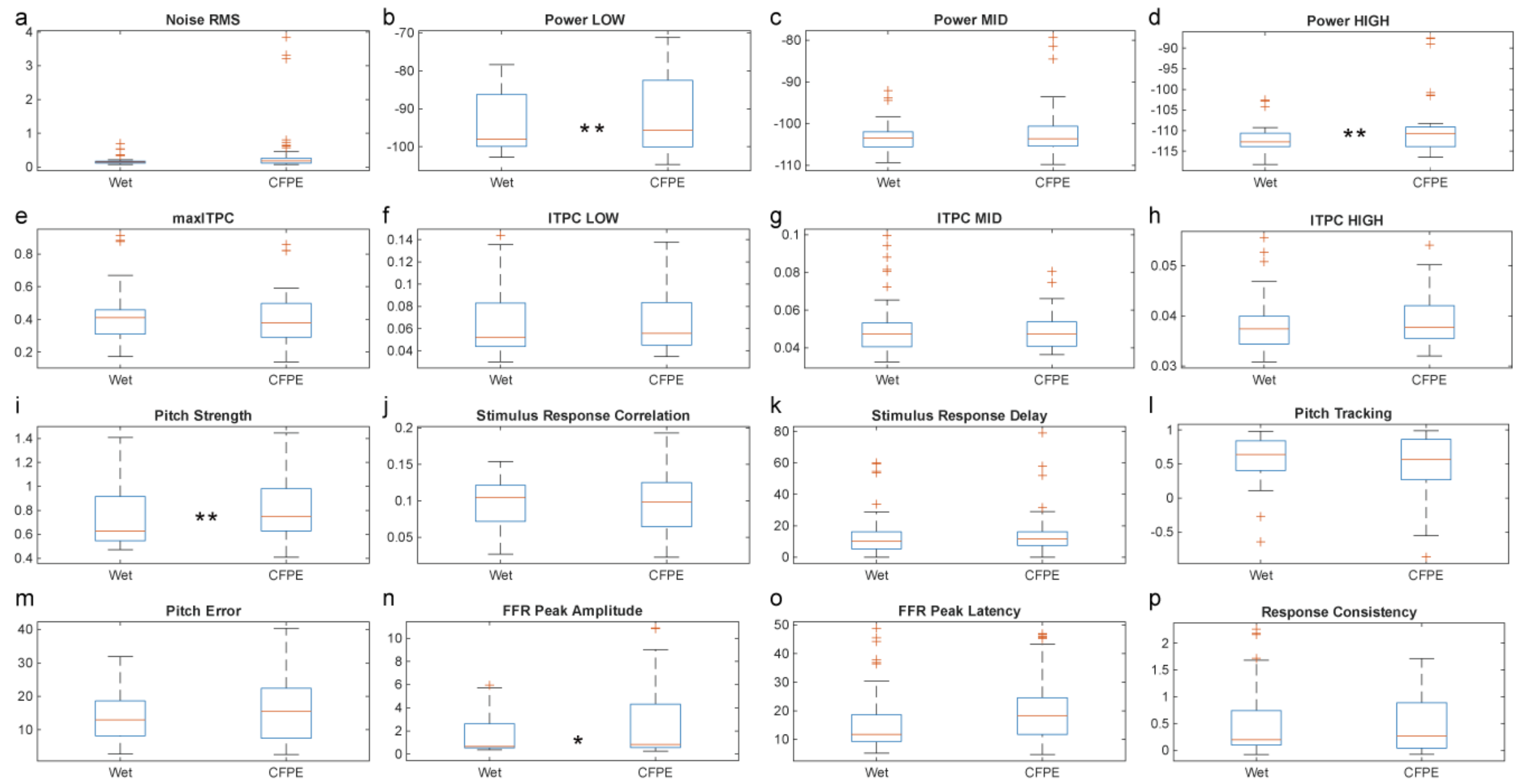

Figure S17. The boxplots of the 16 neural features extracted from the responses recorded with wet (left) and dry electrodes (right). (a) Noise RMS: the pre-stimulus time-domain RMS. (b-d) Power: the frequency-domain FFT power in LOW (120-260 Hz). (b), MID (260-750 Hz). (c) and HIGH (750-1200 Hz). (d) frequency ranges. (e-h) ITPC: inter-trial phase coherence, i.e. the vector average of the magnitude-normalized complex FFT values that captures the degree of phase locking between the individual responses to the same sound, measured as a maximum across its time-frequency representation (max, e) or as a time-domain mean in the LOW (f), MID (g) and HIGH (h) frequency ranges. (i) Pitch Strength: the trough to peak magnitude of the maximal FFR autocorrelation. (j) Stimulus-Response Correlation: the time-domain maximal cross-correlation between the FFR and its eliciting sound. (k) Stimulus-Response Delay: the delay of the FFR waveform in relation to the sound waveform which yields the highest cross-correlation between the two, also known as neural delay. (l) Pitch Tracking: the correlation of the FFR and eliciting sound pitch contours that were extracted from the time-frequency representation of autocorrelation; (m) Pitch Error: the Euclidean distance between the FFR and eliciting sound pitch contours. (n) FFR peak amplitude: the maximal amplitude of the FFR waveform onset, roughly homologous to the peak V of the auditory brainstem response to click. (o) FFR peak latency: the time between the maximal peak and sound onset. (p) Response Consistency: the correlation between sub-averages of the individual trials in the FFR. Statistical comparisons were performed using two-tailed paired t-tests (df = 47), with Benjamini-Hochberg FDR correction across all 18 FFR/LLR features, including FFR SNR and LLR SNR shown in Figure 6. BH-adjusted P values were 0.0044, 0.0070, 0.0070, and 0.0121 for panels b, d, i, and n, respectively; all other comparisons were nonsignificant. *BH-adjusted

P < 0.05; **BH-adjusted P < 0.01; ns, not significant (BH-adjusted P ≥ 0.05). The dataset comprised nine unique participants and 48 tone-level wet-dry paired observations per feature.

Table S1. Summary of independent skin irritation and sensitization assessments of extracts from the CFPE.

Assessment	Standard	Extract vehicle	Model and sample size	Exposure / extraction conditions	Outcome	Result
Multiple-contact skin irritation	GB/T 16886.23-2023	0.9% NaCl, sesame oil	New Zealand White rabbits, n = 3 per extract type	0.2 g/mL, 37 °C, 72 h extraction, 4 h skin contact	Erythema, edema, cumulative irritation index	No erythema or edema, cumulative irritation index = 0 for both extracts
Skin sensitization (GPMT)	GB/T 16886.10-2024	0.9% NaCl, sesame oil	Hartley albino guinea pigs, 20 test animals and 10 negative-control animals	0.2 g/mL, 37 °C, 72 h extraction	Sensitization incidence after challenge	Polar extract: 0%, non-polar extract: 0%, negative control: 0%, positive control: 100%

Skin irritation and sensitization tests were conducted by Guangdong Neway Quality Technology Service Co., Ltd. (China), a CMA-certified testing laboratory (Certificate No. 240015340124) accredited by CNAS (Registration No. L9783). The laboratory holds an experimental animal use license (License No. SYXK [Guangdong] 2024-0160). Skin irritation and sensitization were assessed according to GB/T 16886.23-2023 and GB/T 16886.10-2024, respectively.

Tests were conducted by Guangdong Neway Quality Technology Service Co., Ltd. (China). The finished CFPE device was extracted using both polar and non-polar extraction vehicles to assess the biological response to potentially released constituents under the tested conditions. These tests are biological-response assessments and do not constitute chemical identification or quantification of individual extractables/leachables.

Table S2. Area-normalized equivalent-circuit fitting parameters for the wet electrode and CFPE.

Parameters	Unit	Wet electrode	CFPE
R_1	$\Omega\cdot cm^2$	316.6	48.9
CPE-T	Fs^{P-1}	3.975×10^{-7}	3.144×10^{-7}
CPE-P	$0<P<1$	0.73778	0.60241
R_2	$\Omega\cdot cm^2$	17435	1337
W-R	$\Omega\cdot cm^2$	--	15367
W-T	s	--	1.2654×10^{-3}
W-P	--	--	0.42046

Table S3. Contact impedance at 10 Hz for fresh, durability-challenged, and commercial dry electrodes. Values are presented as mean ± SD. Sample sizes were n = 6 electrodes for fresh CFPEs and commercial dry electrodes and n = 3 electrodes for each durability-challenged condition. The fresh-CFPE baseline in this table was obtained from six electrode specimens in the durability-evaluation series, separately from the three-participant measurements reported in Figure 4c.

**Electrode group**	**Condition**	**Contact impedance at 10 Hz (kΩ·cm²)**
CFPE	Fresh CFPE	11.69 ± 0.70
Commercial dry electrode	Fresh commercial dry electrode	110.95 ± 11.20
CFPE after cyclic compression	4,000 compression cycles	12.13 ± 1.2
	8,000 compression cycles	12.77 ± 0.29
CFPE after repeated application/removal (A/R)	50 A/R cycles	12.11 ± 0.28
	100 A/R cycles	12.56 ± 0.22
CFPE after artificial-sweat cycling	pH 4.7, Cycle 1	10.07 ± 1.92
	pH 4.7, Cycle 25	12.21 ± 0.17
	pH 4.7, Cycle 50	11.93 ± 0.62
	pH 5.5, Cycle 1	11.36 ± 1.33
	pH 5.5, Cycle 25	12.34 ± 0.32
	pH 5.5, Cycle 50	12.53 ± 0.82
	pH 6.5, Cycle 1	10.08 ± 1.03
	pH 6.5, Cycle 25	11.57 ± 1.02
	pH 6.5, Cycle 50	12.27 ± 0.45

Table S4. Signal-to-noise ratio (SNR) comparison between the CFPE and wet electrodes. Values are presented as mean ± SD across three recording runs in one participant for each signal modality. The runs represent repeated measurements rather than independent participants.

Signal type	Wet electrode, SNR (dB)	CFPE, SNR (dB)
ECG	23.25 ± 0.74	21.23 ± 0.41
EMG	31.10 ± 2.38	34.76 ± 2.23
EEG	13.07 ± 1.39	11.58 ± 2.19

Table S5. Descriptive low-frequency ECG fluctuation RMS values during seated rest and slow walking. Values are presented as mean ± SD across three experimental runs in one participant, using a different electrode specimen for each condition in each run. RMS was calculated from the 0.05-0.5 Hz component of the ECG recordings. The comparison is descriptive.

Electrode condition	Rest RMS, (μV)	Walking RMS, (μV)
Fresh wet electrode	170.34 ± 68.72	698.28 ± 345.16
Fresh CFPE	202.43 ± 133.65	518.26 ± 265.74
CFPE after 8,000 compression cycles	177.29 ± 76.96	579.21 ± 299.91
CFPE after 100 application/removal cycles	200.73 ± 95.45	667.92 ± 331.63
CFPE after 50 artificial-sweat cycles at pH 4.7	190.57 ± 91.26	687.96 ± 328.59
Commercial dry electrode	493.38 ± 262.21	793.44 ± 370.75

Table S6. Statistical comparison of the 18 FFR/LLR features between wet electrodes and CFPEs. The dataset included nine unique participants, seven of whom completed an additional reverse-order session, resulting in 16 sessions and 48 tone-level wet-dry paired observations per feature. Two-tailed paired t-tests were performed using these 48 pairs (df = 47). Unadjusted P values and P values adjusted using the Benjamini-Hochberg procedure across all 18 features are reported. Significance symbols refer to BH-adjusted P values: *P < 0.05; **P < 0.01; ns, P ≥ 0.05.

FEATURE	T-STATISTIC	DF	UNADJUSTED P VALUE	BH-ADJUSTED P VALUE	SIGNIFICANCE
FFR SNR	0.047915	47	0.961987	0.961987	ns
NOISE RMS	−2.463130	47	0.017491	0.052474	ns
POWER LOW	−3.747300	47	0.000488	0.004395	**
POWER MID	−2.242040	47	0.029717	0.066864	ns
POWER HIGH	−3.412000	47	0.001335	0.007018	**
MAXITPC	0.801258	47	0.427015	0.591251	ns
ITPC LOW	−0.239340	47	0.811883	0.859640	ns
ITPC MID	1.336975	47	0.187666	0.307090	ns
ITPC HIGH	−1.061750	47	0.293775	0.440662	ns
PITCH STRENGTH	−3.358900	47	0.001560	0.007018	**
STIMULUS RESPONSE CORRELATION	−0.343240	47	0.732949	0.859640	ns
STIMULUS RESPONSE DELAY	−0.245150	47	0.807408	0.859640	ns
PITCH TRACKING	1.534739	47	0.131553	0.236795	ns
PITCH ERROR	−1.925840	47	0.060186	0.120371	ns
FFR PEAK AMPLITUDE	−3.090560	47	0.003353	0.012071	*
FFR PEAK LATENCY	−2.301480	47	0.025844	0.066457	ns
RESPONSE CONSISTENCY	0.366787	47	0.715424	0.859640	ns
LLR SNR	−3.897470	47	0.000307	0.004395	**

Table S7. Comparison of state-of-the-art flexible dry electrodes.

Electrodes	Skin Preparation	Stability	Microvolt-Level EEG Acquisition	Interfacial Impedance	Signal Type	Reference
CFPE	No	8-week storage stability	Yes	11.09 kΩ·cm^2 @ 10 Hz	EEG/ECG/EMG	This work
PEDOT:PSS/WPU	No	1 month	Yes	82 kΩ·cm^2 @10 Hz	EEG/ECG/EMG	[1]
PEDOT:PSS/PVA	No	30 days	No	77 kΩ·cm^2 @20 Hz	ECG/EMG	[2]
MXene/PVDF LBNF	Yes	10 days	No	107.8 kΩ·cm^2 @10 Hz	ECG	[3]
Galinstan/PDMS (S-LMC)	No	7 days	No	7.35 kΩ·cm^2 @10 Hz	ECG	[4]
Mycelium/PPy (P-MCM)	No	3 months	Yes	52.6 kΩ·cm^2 @10 Hz	EEG/ECG/EMG	[5]
PEDOT:PSS/PVA/TP (CPT)	No	72 h	No	14 kΩ·cm^2 @100 Hz	EMG/ECG	[6]

References

1. L. Zhang, K.S. Kumar, H. He, et al., "Fully organic compliant dry electrodes self-adhesive to skin for long-term motion-robust epidermal biopotential monitoring," *Nat Commun* 11 (2020): 4683, https://doi.org/10.1038/s41467-020-18503-8.

2. S. Hou, D. Lv, Y. Li, et al., "Highly Stretchable Self-Adhesive PEDOT:PSS Dry Electrodes for Biopotential Monitoring," *ACS Appl Polym Mater* 6 (2024): 7781-7793, https://doi.org/10.1021/acsapm.4c01233.

3. M.A. Zahed, D.K. Kim, S.H. Jeong, et al., "Microfluidic-Integrated Multimodal Wearable Hybrid Patch for Wireless and Continuous Physiological Monitoring," *ACS Sens* 8 (2023): 2960-2974, https://doi.org/10.1021/acssensors.3c00148.

4. S.W. Lee, H. Song, J. Kim, et al., "Self-Adhesive Liquid Metal Channel Patch with Tip-Guided Conformal Coupling and Leakage Suppression for Skin Bioelectronics," *Advanced Science* 13 (2026): e13259, https://doi.org/10.1002/advs.202513259.

5. R. Zhang, S. Cheng, Z. Pan, et al., "Biovegan Leather Sensor: A Mycelium Functionalized Material for Electrophysiological Signal Monitoring," *ACS Appl Mater Interfaces* 17 (2025): 34538-34547, https://doi.org/10.1021/acsami.5c05377.

6. S. Yang, C. Liu, L. Tang, J. Shang, J. Zhang, X. Jiang, "Highly Adhesive and Stretchable Epidermal Electrode for Bimodal Recording Patch," *ACS Appl Mater Interfaces* 16 (2024): 43880-43891, https://doi.org/10.1021/acsami.4c05705.
